# Safety of rTMS for Schizophrenia: A Systematic Review and Meta-analysis

**DOI:** 10.1093/schbul/sbae158

**Published:** 2024-09-15

**Authors:** Sophia H Blyth, Claudia Cruz Bosch, Julian J Raffoul, Jordyn Chesley, Benjamin Johnson, Darara Borodge, Raza Sagarwala, Ross Masters, Roscoe O Brady, Simon Vandekar, Heather Burrell Ward

**Affiliations:** Department of Psychiatry and Behavioral Sciences, Vanderbilt University Medical Center, Nashville, TN, USA; Department of Psychiatry and Behavioral Sciences, Vanderbilt University Medical Center, Nashville, TN, USA; Department of Psychiatry and Behavioral Sciences, Vanderbilt University Medical Center, Nashville, TN, USA; Department of Psychiatry and Behavioral Sciences, Vanderbilt University Medical Center, Nashville, TN, USA; Department of Psychiatry and Behavioral Sciences, Vanderbilt University Medical Center, Nashville, TN, USA; Department of Psychiatry and Behavioral Sciences, Vanderbilt University Medical Center, Nashville, TN, USA; Department of Psychiatry and Behavioral Sciences, Vanderbilt University Medical Center, Nashville, TN, USA; Department of Psychiatry and Behavioral Sciences, Vanderbilt University Medical Center, Nashville, TN, USA; Department of Psychiatry, Harvard Medical School and Beth Israel Deaconess Medical Center, Boston, MA, USA; McLean Hospital, Belmont, MA, USA; Department of Biostatistics, Vanderbilt University Medical Center, Nashville, TN, USA; Department of Psychiatry and Behavioral Sciences, Vanderbilt University Medical Center, Nashville, TN, USA

**Keywords:** schizophrenia, transcranial magnetic stimulation, safety, adverse effects, seizure, neuromodulation

## Abstract

**Background and Hypothesis:**

Current treatments for schizophrenia are only partially effective, and there are no medications for negative symptoms or cognitive impairment. Neuromodulation, such as repetitive transcranial magnetic stimulation (rTMS), has potential as a novel intervention for schizophrenia. Prior to clinical use, rTMS should have demonstrated safety in a large schizophrenia population. However, the safety profile of rTMS in schizophrenia is not well characterized, and regulatory agencies have expressed concern about safety in this population.

**Study Design:**

We conducted a systematic review with meta-analysis of rTMS studies in schizophrenia. We searched PubMed, the Cochrane Library, PsycINFO, and Science Citation Index Expanded for rTMS studies in schizophrenia that reported adverse effects. We extracted the number of participants who experienced an adverse effect and calculated the prevalence of each adverse effect for active or sham rTMS. We tested the difference between the prevalence of events in the active and sham conditions. We assessed risk of bias using the Cochrane Handbook.

**Study Results:**

The initial search identified 1472 studies. After screening, 261 full-text studies were assessed, and 126 met inclusion criteria (*N* = 4122 total subjects). The prevalence of headache or scalp pain, dizziness or syncope, facial twitching, and nausea was higher for active rTMS compared to sham (*P* < .05). The prevalence of all other adverse effects, including seizure, was not different between active and sham rTMS.

**Conclusions:**

rTMS is safe and well tolerated for people with schizophrenia. Individuals with schizophrenia are not at increased risk for adverse effects, including seizure, compared to the general population.

## Introduction

Schizophrenia is a chronic psychotic illness and leading cause of disability worldwide.^1^ Schizophrenia is characterized by delusions, hallucinations, disorganized speech and behavior, negative symptoms, and cognitive impairment.^2^ Treatment involves use of antipsychotic medication and psychotherapy. However, antipsychotics are only partially effective, substantially improving symptoms in less than 25% of individuals,^3^ and only a minority of individuals achieve remission.^4^ Antipsychotics are most effective for positive symptoms, and there are no current pharmacologic treatments for negative symptoms or cognitive impairment in schizophrenia. Given the limited treatment options for schizophrenia, the field has frantically searched for alternative interventions.

Neuromodulation, or the use of brain stimulation to change neuronal activity, is one such intervention that has been explored for schizophrenia. Repetitive transcranial magnetic stimulation (rTMS) is a form of noninvasive neuromodulation that involves pulsing an electrical current through an external electromagnetic coil placed on the scalp and using the induced electromagnetic field to temporarily alter patterns of neuronal activity. rTMS is approved by the Food and Drug Administration (FDA) for treatment of major depressive disorder (MDD),^5^ obsessive compulsive disorder (OCD),^6^ and smoking cessation.^7^ Since its initial FDA approval for MDD in 2008, rTMS has become a valuable and readily available research tool to probe neural circuitry involved in many other psychiatric disorders, including schizophrenia, where it has been studied as an intervention for auditory hallucinations, negative symptoms, and cognitive performance, among others. In Europe, rTMS has a *conformité européenne* mark (ie, European version of FDA clearance) for treatment of negative symptoms in schizophrenia.^8^ rTMS is generally very well tolerated, as the most common side effect is headache. In contrast to interventions like electroconvulsive therapy, rTMS does not involve the use of anesthesia and has no cognitive side effects. Because rTMS stimulates neurons to depolarize, rTMS is associated with risk of seizure. When international consensus guidelines are followed,^9,10^ rTMS has a seizure risk of less than 1/1000.^11,12^

However, there are questions about the scalability of rTMS. Is rTMS safe and appropriate for use in large populations of people with schizophrenia? Although there have been many studies and multiple systematic reviews of rTMS in schizophrenia,^13–16^ some regulatory agencies have expressed concern about its safety in this population due to the illness itself (ie, does rTMS worsen psychotic or mood symptoms) and use of medications that may alter seizure threshold,^17^ thereby increasing risk of rTMS-induced seizure. Importantly, no one has ever characterized the safety profile of rTMS in schizophrenia. We therefore conducted a systematic review with meta-analysis to characterize the safety and adverse event profile of rTMS in schizophrenia spectrum disorders. We hypothesized that there would be no differences in the prevalence of serious adverse events between active and sham rTMS in schizophrenia and that the prevalence of seizure would be at or below the prevalence in the general population.

## Methods

### Protocol and Registration

This systematic review is reported in accordance with the Preferred Reporting Items for Systematic Review and Meta-Analyses (PRISMA) Statement^18^ and is registered in the PROSPERO database (http://www.crd.york.ac.uk/PROSPERO/) under the number CRD42022367460.

### Eligibility Criteria

Articles that used rTMS for adults with schizophrenia spectrum disorders were included. Studies involving children (<18 years old) were excluded. Studies comparing rTMS with a control treatment (sham or no treatment) were included, but not all studies had a comparison group. The search strategy included descriptors for rTMS and schizophrenia spectrum disorders (Supplemental Table 1). Randomized controlled trials (RCTs), cohort studies, case-control studies, cross-sectional studies, case reports, case series, and systematic chart reviews published in peer-reviewed journals, and letters to the editor were considered for this systematic review. Conference abstracts, literature reviews, systematic reviews, and meta-analyses were excluded. Publications without safety or adverse event outcomes were also excluded.

### Primary Outcome

The primary outcome for this study was the prevalence of adverse effects associated with rTMS in individuals with schizophrenia. A priori, we defined adverse effects as seizure; headache or scalp pain; neck pain; dizziness or syncope; fatigue; cognitive impairment; worsening psychosis; worsening depression; and worsening mania. In the course of our review, multiple studies also reported nausea, facial twitching, and insomnia, so data on these adverse effects were also extracted and included in our analysis.

### Search Strategy

We searched the following online databases: PubMed, the Cochrane Library, PsycINFO EBSCOhost, (1872 to present), and Science Citation Index Expanded (ISI Web of Science, 1945 to present) on November 22, 2022. We utilized a wide-ranging search strategy using broad search terms with the goal of including all studies of rTMS in schizophrenia. The initial search contained the MeSH terms “Schizophrenia Spectrum and Other Psychotic Disorders” and “Transcranial Magnetic Stimulation,” as well as synonyms for schizophrenia or schizoaffective disorder and TMS. We also included search terms to exclude animals, infants, children and adolescents, and certain publication types (review, systematic review, or meta-analysis). There were no restrictions on publication date or language. The detailed search strategy for each of the databases is shown in Supplemental Table 1.

### Study Selection

**Fig. 1.**
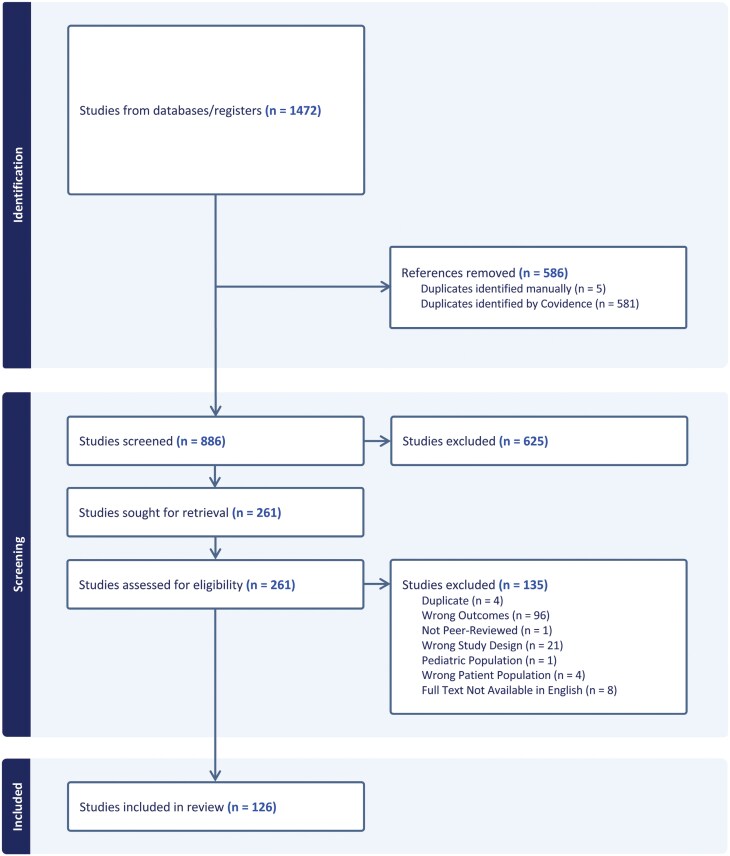
PRISMA diagram. We performed a comprehensive literature search to identify all studies testing repetitive transcranial magnetic stimulation (rTMS) in schizophrenia. Of an initial 1472 studies, we identified 126 studies that met criteria for inclusion in this review and meta-analysis.

Titles and abstracts of the retrieved articles were independently evaluated by two reviewers (J.R., J.C.). Studies clearly not meeting inclusion criteria were excluded based on title and abstract. The remaining studies were assessed based on full-text articles and selected if they fully met the inclusion and exclusion criteria (figure 1). All screens were performed by two separate reviewers (J.C., J.R.) with discrepancies resolved by a third reviewer (H.B.W.). Duplicate publications and duplicate data were removed. The search strategy is shown in Supplemental Table 1. Following this initial search and screen, we manually searched the references and performed a citation analysis of the included studies to identify any additional articles that met inclusion criteria.

### Data Extraction

Seven reviewers (C.C.B., J.R., J.C., B.J., D.B., R.S., R.M.) independently conducted the data extraction. Two reviewers extracted data for each article, and disagreements were resolved by a third reviewer (H.B.W.). General characteristics of the studies were collected, such as author, year of publication, location of study, study design, study population, sample size, and rTMS treatment parameters (target, frequency/montage, pulses/session, number of sessions, neuronavigation, and coil shape). For the primary outcome (prevalence of adverse effects), we defined adverse effects a priori as seizure; headache or scalp pain; neck pain; dizziness or syncope; fatigue; cognitive impairment; worsening psychosis; worsening depression; and worsening mania. Of these, we defined serious adverse effects a priori to include: seizure, cognitive impairment, or worsening psychosis, depression, or mania. We extracted the number of participants who experienced each adverse effect for the active and sham groups (where applicable). We also collected any other adverse effects reported by each study. For studies that reported “no serious adverse effects were observed,” we reported zero for each predefined serious adverse event category.

### Statistical Analysis

We combined data across all studies by summing the total sample sizes and total number of events, which assumes that participants are independent within and across studies. For each adverse event, we used the Clopper-Pearson exact confidence interval for the proportion, and we tested the difference between proportion of events in the active and sham conditions using Pearson’s chi-square test or Fisher’s exact test when the number of events in either the sham or active group was less than five.

### Quality Assessment

Assessment of methodologic quality of the studies and risk of bias was performed for each included study as suggested by the Cochrane Handbook^18^ and Cochrane Risk of Bias tools (methods.cochrane.org): (1) *high risk* when more than one indicator of bias was present across all scales and (2) *low risk* when one or no indicator was present. The risk of bias was classified by seven independent reviewers (J.R., J.C., C.C.B., B.J., D.B., R.S., R.M.), and differences were resolved by a third reviewer (H.B.W.). No studies were excluded based on degree of risk of bias.

## Results

### Study Characteristics

A total of 1472 studies were identified by conducting a database search in PubMed, PsycINFO, the Cochrane Library, and Embase. After removing duplicates, irrelevant studies, and those meeting exclusion criteria, 126 studies were included in the analysis (figure 1). Studies followed various study designs, including case reports (*n* = 18), nonrandomized experimental studies (*n* = 23), RCTs (*n* = 80), case series (*n* = 4), and cohort studies (*n* = 1). The studies were conducted in different countries of origin, including India (*n* = 15), China (*n* = 14), Germany (*n* = 12), France (*n* = 11), Netherlands (*n* = 11), USA (*n* = 12), Czech Republic (*n* = 10), Canada (*n* = 8), Australia (*n* = 6), South Korea (*n* = 6), Israel (*n* = 5), Switzerland (*n* = 4), Brazil (*n* = 3), United Kingdom (*n* = 2), Belgium (*n* = 1), Chile (*n* = 1), Finland (*n* = 1), Iran (*n* = 1), Spain (*n* = 1), and Sweden (*n* = 1). One study did not report the country of origin. The quality of included studies was highly variable, given the inclusion of case reports, other unblinded studies, and rTMS studies that did not use a sham coil. See Supplemental Tables 2 and 3.

The study population consisted of 4122 participants, of which 2152 received active rTMS (two studies did not report the number of participants who did not receive active rTMS) and 1358 received sham rTMS (48 studies either did not use sham rTMS or did not report the number of participants who received sham rTMS). Each study included an average of 33 participants (SD = 52). On average, 17 participants per study received active rTMS (SD = 18), and 17 participants per study received sham rTMS (SD = 13). Individuals who received active rTMS ranged in age from 20 to 53 years old, and individuals who received sham rTMS ranged from 22 to 56 years old. The average age of participants who received active rTMS was 37.16 years old (calculated from 104 studies). The average age of participants who received sham rTMS was 37.86 years old (calculated from 71 studies). All participants had a diagnosis of schizophrenia or schizoaffective disorder, although some had comorbid MDD, OCD, or cannabis use disorder. Among the individuals who received active rTMS, an average of 29.92% of these participants were female (calculated from 110 studies). Among the individuals who received sham rTMS, an average of 30.28% of these participants were female (calculated from 69 studies). Various ethnic groups were represented in the patient population, including African Americans, Caucasians, Asians, Pacific Islanders, Hispanics/Latinos, and others.

Participants in the included studies were generally taking antipsychotic medication. The average chlorpromazine equivalents (CPZeq) for active rTMS participants was 484.17 (calculated from 36 studies) (range 184.21–1168). The average CPZeq among individuals who received sham rTMS was 515.49 (197.22–1309) (calculated from 35 studies). Both active and sham groups included individuals on clozapine monotherapy or dual antipsychotic therapy with clozapine and other antipsychotic medications. An average of 32.27% of people receiving active rTMS were on clozapine (range 0–100; calculated from 59 studies), and 32.54% of people receiving sham rTMS were on clozapine (range 0–100; calculated from 32 studies). Approximately 286 people who received active rTMS were taking clozapine, while approximately 179 people who received sham rTMS were taking clozapine.

The number of rTMS sessions varied by study, with an average of 14.98 active rTMS sessions (range 1–90; calculated from 119 studies). Most studies used a figure-of-eight coil (*n* = 98), with some using a 9 cm circular coil (*n* = 3), 14 cm circular coil (*n* = 1), H1 coil (*n* = 2), H4 coil (*n* = 1), unspecified circular coil (*n* = 4), unspecified H coil (*n* = 1), angled double-coned coil (*n* = 1), and an unspecified type of coil (*n* = 14). One study used a figure-of-eight for 10 Hz and 20 Hz rTMS, and a circular coil for iTBS. There was substantial heterogeneity in the sham procedures used: sham coil (*n* = 32), similar coil titled 45–90°C off the scalp or flipped 180°C (*n* = 42), or stimulation over the occipital cortex as an active comparator (*n* = 2). An active coil was used in one of the sham-protocol studies but placed 2ft behind the participant with an unplugged coil placed in the participant’s forehead. Forty-nine studies did not use a control protocol. Treatment intensity ranged from 80% to 130% motor threshold (MT). Frequencies of rTMS included were 0.9–1 Hz (*n* = 53), 10 Hz (*n* = 33), 20 Hz (*n* = 22), iTBS (*n* = 12), cTBS (*n* = 10), 5–7 Hz (*n* = 2), 8–13 Hz (*n* = 1), 18 Hz (*n* = 1), 15 Hz (*n* = 1), 3 Hz (*n* = 1), and 0.033 Hz (*n* = 1). Two studies did not specify the frequency of rTMS applied.

Various primary symptoms were targeted, including auditory hallucinations (*n* = 51), negative symptoms (anhedonia, avolition, and blunted affect) (*n* = 42), neurocognitive functioning including working and visuospatial memory (*n* = 19), general schizophrenia or psychotic symptoms (*n* = 10), positive symptoms (*n* = 9), depressive symptoms (*n* = 6), smoking behavior/tobacco craving (*n* = 5), cortical inhibition (*n* = 3), OCD symptoms (*n* = 2), motor cortical excitability (*n* = 2), electroencephalogram (EEG) changes (*n* = 2), general psychiatric symptoms (assessed by Brief Psychiatric Rating Scale, *n* = 2), anxiety symptoms (*n* = 2), visual hallucinations (*n* = 1), resting MT changes (*n* = 1), first-rank symptoms (*n* = 1), cannabis use (*n* = 1), hand gesture deficits (*n* = 1), psychomotor activity (*n* = 1), global improvement (*n* = 1), weight loss (*n* = 1), brain structure (*n* = 1), and the causal connectivity between the dorsolateral prefrontal cortex (DLPFC) and inferior parietal lobule (IPL, *n* = 1). In the active rTMS protocols, various brain regions were targeted, including the left, right, or bilateral temporal or temporoparietal cortex (TPC) (*n* = 56); the left, right, or bilateral DLPFC (*n* = 47); the left, right, or bilateral prefrontal cortex (PFC) (*n* = 9); the cerebellar vermis (*n* = 7); the motor or supplemental motor cortex (*n* = 4); the ventrolateral prefrontal cortex (VLPFC) (*n* = 3); the occipital cortex (*n* = 2); the bilateral dorsomedial prefrontal cortex (DMPFC) (*n* = 2); and the bilateral insula (*n* = 1). Approximately 22% of studies (28 studies) used neuronavigation to target specific brain areas.

### Meta-analysis: Prevalence of Side Effects

**Fig. 2.**
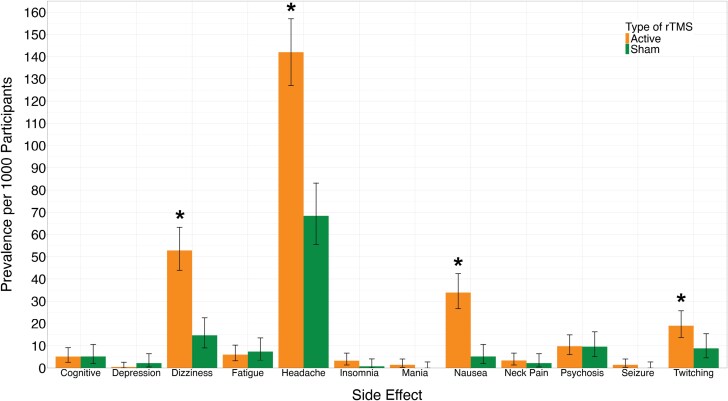
Prevalence of rTMS side effects in schizophrenia. We calculated the prevalence of each adverse effect for active rTMS treatment and for sham rTMS. The prevalence of headache or scalp pain, dizziness or syncope, facial twitching, and nausea were significantly higher in active rTMS compared to sham (*P < .05). The calculated prevalence of each other adverse effect, including seizure, was not different between active and sham rTMS. rTMS, repetitive transcranial magnetic stimulation.

After extracting the adverse effects reported in each study, we calculated the prevalence of each reported side effect across all included studies. We calculated the prevalence of each adverse effect for active rTMS treatment and for sham rTMS, shown in table 1 and figure 2. The prevalence of headache or scalp pain; dizziness or syncope; facial twitching; and nausea were significantly higher in active rTMS compared to sham (*P* < .05). The calculated prevalence of each other adverse effect, including seizures, was not different between active and sham rTMS (table 1 and figure 2).

**Table 1.** Estimated Prevalence of Side Effects

Side Effect	Active rTMS (Estimate, 95% CI)	Sham rTMS (Estimate, 95% CI)	*P*
Seizure	1.39/1000 (0.287/1000–4.07/1000)	0.00/1000 (0.00/1000–2.71/1000)	.288
Headache or scalp pain	142/1000 (127/1000–157/1000)	68.4/1000 (55.5/1000–83.1/1000)	<.0001*
Neck pain	3.25/1000 (1.31/1000–6.69/1000)	2.21/1000 (0.455/1000–6.43/1000)	.750
Dizziness or syncope	52.9/1000 (43.9/1000–63.3/1000)	14.7/1000 (9.01/1000–22.6/1000)	<.0001*
Fatigue	6.04/1000 (3.22/1000–10.3/1000)	7.35/1000 (3.53/1000–13.5/1000)	.798
Cognitive impairment	5.11/1000 (2.55/1000–9.12/1000)	5.15/1000 (2.07/1000–10.6/1000)	1
Worsening psychosis	9.75/1000 (6.05/1000–14.9/1000)	9.56/1000 (5.10/1000–16.3/1000)	1
Worsening depression	0.464/1000 (0.0118/1000–2.59/1000)	2.21/1000 (0.455/1000–6.43/1000)	.306
Worsening mania	1.39/1000 (0.287/1000–4.07/1000)	0.00/1000 (0.00/1000–2.71/1000)	.288
Facial twitching	19.0/1000 (13.7/1000–25.7/1000)	8.82/1000 (4.57/1000–15.4/1000)	.023*
Nausea	33.9/1000 (26.7/1000–42.4/1000)	5.15/1000 (2.07/1000–10.6/1000)	<.0001*
Insomnia	3.25/1000 (1.31/1000–6.69/1000)	0.735/1000 (0.0186/1000–4.10/1000)	.162

After identifying all studies testing repetitive transcranial magnetic stimulation (rTMS) interventions in schizophrenia, we extracted the number of participants who experienced each adverse effect for the active and sham groups and calculated the estimated prevalence and confidence interval for each side effect.

We identified a total of three seizures in people with schizophrenia.^19–21^ All seizures occurred with rTMS administered at 20 Hz at^19,20^ or above MT.^21^ One seizure occurred with an H-coil^21^ (known to have a higher risk of seizure^11^), and two occurred with figure-of-eight coils.^19,20^ One seizure occurred in a patient taking clozapine 500 mg daily,^20^ although 285 other participants taking clozapine did not have seizures. The prevalence of seizure among individuals taking clozapine was 3.49/1000 (95% CI 0.0885/1000–19.3/1000), which was not significantly different (*P* > .05) from the prevalence of seizure in the overall schizophrenia population (1.39/1000; 95% CI 0.288/1000–4.07/1000, table 1). We did not identify any seizures caused by iTBS, cTBS, or low frequency rTMS.

Given the substantial heterogeneity in rTMS parameters, we also calculated the prevalence of side effects for each rTMS target and stimulation protocol (see Supplemental Tables 4–17). For each stimulation target, we compared the prevalence of side effects with active rTMS at that target to the side effect prevalence with any sham stimulation. To compare side effects between stimulation protocols, we grouped stimulation by high frequency, low frequency, iTBS, or cTBS. For each stimulation group, we compared the prevalence of side effects with active rTMS for a given stimulation protocol to the side effect prevalence with any sham stimulation. We included studies that tested two active targets or two active protocols if they reported the side effects for each specific target or protocol. Six studies administered rTMS to multiple brain targets.^22–27^ We excluded four of these studies from analysis, as they did not report side effect by target, administered rTMS to the same brain region multiple times and at different frequencies, or administered rTMS at more than one brain region to all participants.^22,25–27^ Twelve studies administered rTMS at multiple frequencies.^22,23,26–35^ We excluded five of these studies from analysis, as they employed a priming protocol of two frequencies at once, did not report side effects by treatment target, or administered more than one frequency to all participants.^26–28,32,35^

Overall, the side effect profiles by rTMS target and stimulation protocol were similar to the overall comparisons of active vs sham rTMS (see Supplemental Tables 4–17). However, active rTMS to some targets was associated with a higher prevalence of specific side effects compared to sham (*P* < .05): temporal lobe/TPC: cognitive impairment; DLPFC: insomnia; cerebellar vermis: worsening mania; and VLPFC: fatigue. Likewise, some active stimulation protocols were associated with a higher prevalence of specific side effects compare to sham (*P* < .05): low frequency: cognitive impairment; iTBS: fatigue and worsening mania; and cTBS: neck pain.

## Discussion

Research using rTMS for schizophrenia has grown rapidly over the past several decades^36^ and there is preliminary evidence that rTMS may be an effective treatment for multiple symptom domains in schizophrenia.^13–16^ As rTMS research in schizophrenia is expanding and seeks to be translated into the clinic, there are questions about its safety when applied on a large scale. Although rTMS is generally well tolerated in psychiatric populations, the safety and adverse effect profile of rTMS has not been quantified for schizophrenia. Traditionally, rTMS adverse effects are quantified based on surveys of clinical providers.^11^ Given rTMS is not used clinically for schizophrenia, our analysis used a novel meta-analytic approach to characterize and quantify the adverse effect profile of rTMS in schizophrenia spectrum disorders.

In our systematic review, we identified 126 studies that tested rTMS in a total of 4122 participants with schizophrenia spectrum disorders. When we compared the prevalence of adverse effects between active and sham treatment, headache or scalp pain, dizziness or syncope, facial twitching, and nausea were more prevalent with active compared to sham rTMS (*P* < .05). This is to be expected, as these are all well-documented side effects of rTMS in the general population. Overall, the prevalence of adverse effects we observed in schizophrenia was at or below the prevalence reported in the general population. For example, the prevalence of headache or scalp pain was 14% in schizophrenia, compared to estimates of approximately 20%–40% in the general population.^37^

We did not observe any evidence of worsening psychiatric symptoms, including psychosis, or cognitive impairment with rTMS administration in schizophrenia. Although caution should be exercised whenever testing an intervention for individuals with psychiatric disorders, there should be little concern that rTMS will worsen mood or psychosis symptoms in schizophrenia. An important consideration is that our analysis pooled all active rTMS interventions, regardless of rTMS stimulation site. With increasing evidence for symptom-specific circuit modulation in schizophrenia,^38^ rTMS benefits and side effects are likely dependent on the target, circuit, and interaction between the two. We compared the prevalence of side effects for each rTMS target and stimulation protocol and observed some minor differences. However, these results should be viewed as exploratory. Given the wide range of rTMS parameters and limited number of studies, our analysis was not powered to compare side effects between stimulation protocols at the same rTMS target. This would be important for future analyses.

We identified a total of three seizures in people with schizophrenia,^19–21^ all with 20 Hz rTMS. We did not identify any seizures caused by iTBS, cTBS, or low frequency rTMS. Our calculated prevalence of seizure in schizophrenia with active rTMS was 1.39/1000 patients (95% CI 0.287/1000–4.07/1000), which was not different from the prevalence of seizure with sham rTMS. This is comparable to the risk of seizure with rTMS observed in the general population, which is estimated to be approximately 1/1000 patients. A survey study of patients receiving clinical rTMS treatment performed by Taylor et al observed an overall seizure prevalence of 18 seizures per 25 526 patients, or 0.71/1000,^11^ which is included in the confidence interval of our estimate. Importantly, the seizure prevalence varied between devices, ranging up to 5.56 seizures per 1000 patients for Brainsway devices, which use H-coils. For comparison, the prevalence of unprovoked seizure in the general population aged 18–69 is also estimated to be 1/1000.^39^ As another comparison, a recent study of the risk of MRI-related incidents observed 921 MRI safety incidents of 312 288 MRIs (a prevalence of 29/1000).^40^ Therefore, the risk of rTMS-induced seizure in schizophrenia is comparable to the prevalence of seizure in the general population, and the prevalence of adverse events with common medical procedures (ie, MRI) that are not considered significant risk.

There have also been concerns that psychiatric medications that lower seizure threshold, such as clozapine, may be associated with increased risk of rTMS-induced seizure. In our analysis, the prevalence of seizure was not different between individuals taking clozapine and the overall schizophrenia population. This is consistent with the latest international TMS consensus guidelines,^9,10^ which state no psychiatric medication or psychiatric disorder increases the risk of side effects from rTMS.

Importantly, individuals with schizophrenia have an increased prevalence of epilepsy. The incidence of epilepsy in schizophrenia is estimated to be four to five times that of the general population.^41^ However, this should not imply that individuals with schizophrenia will be at increased risk of rTMS-induced seizure. Our analysis did not identify any increased risk of rTMS-induced seizure in participants with schizophrenia. This suggests that there is no indication for more rigorous screening for seizure risk or more stringent regulatory protocols for rTMS in schizophrenia than are recommended for the general population.^9,10,42^

Our analysis also highlights the persistent challenge in determining safety and adverse effect profiles for rTMS. Given the very low prevalence of seizure, any single clinical trial is unlikely to observe a seizure, so systematic reviews and meta-analyses are the only ways to estimate safety profiles of rTMS in research studies. Accordingly, it is critical that adverse effects are accurately reported in rTMS research studies, including details such as the adverse effect observed, number of events, whether the participant received active or sham stimulation, and relationship to rTMS. In our analysis, we excluded 96 studies because they did not report any adverse effects or safety information. Therefore, the prevalence of adverse effects with rTMS in schizophrenia we calculated in our analysis is likely an overestimate. Moreover, research studies may use higher risk rTMS protocols in higher risk populations, so the collection of adverse effects in clinical treatment is a critical comparator. Given rTMS is not currently used for clinical treatment of schizophrenia, these numbers do not exist for this population. To accurately determine the prevalence of adverse effects with clinical treatment, others have administered surveys of clinical practitioners,^11^ which will continue to be valuable for FDA-cleared indications.

In summary, rTMS is a clinical and research tool being increasingly used to study and treat schizophrenia among other psychiatric disorders. In this systematic review and meta-analysis, we determined that rTMS is a safe and well-tolerated intervention for people with schizophrenia and that individuals with schizophrenia are not at increased risk for adverse effects, including seizure, compared to the general population. Additional safety screening and regulatory procedures are likely unwarranted and may do more harm by slowing the pace of psychiatric research for a vulnerable population and excluding those individuals from research who need it most. Future research studies should ensure detailed information are captured about adverse effects that occur with rTMS to improve the accuracy of our risk assessments and safety for our participants.

## Supplementary Material

Supplementary material is available at https://academic.oup.com/schizophreniabulletin/.

sbae158_suppl_Supplementary_Tables_1-17
