# Supplementary material for: Safety of rTMS for Schizophrenia: A Systematic Review and Meta-analysis
Source: Schizophr Bull. 2024 Sep 15;51(2):392–400. doi: 10.1093/schbul/sbae158 (PMC11908856; doi:10.1093/schbul/sbae158)
Supplement: sbae158_suppl_Supplementary_Tables_1-17 [file sbae158_suppl_supplementary_tables_1-17.docx]

**Supplemental Table 1. Search Strategy­­­**

| **PubMed** | | |
| --- | --- | --- |
| 1 | "Schizophrenia Spectrum and Other Psychotic Disorders"[Mesh] | 160316 |
| 2 | schizophreni*[tiab] | 140179 |
| 3 | schizoaffective[tiab] | 6491 |
| 3 | 1 OR 2 OR 3 | 202815 |
| 4 | "Transcranial Magnetic Stimulation”[MeSH] | 14290 |
| 5 | (“transcranial magnetic stimulation”[tiab] | 17961 |
| 6 | “theta burst stimulation”[tiab] | 1844 |
| 7 | 4 OR 5 OR 6 | 21182 |
| 8 | 3 AND 7 | 732 |
| 9 | NOT ("animals"[MeSH]) | 105 |
| 10 | NOT ("child"[MeSH] OR "adolescent"[MeSH] OR "infant"[MeSH]) | 105 |
| 11 | NOT ("Review"[Publication Type] OR "Systematic Review"[Publication Type] OR "Meta-Analysis"[Publication Type]) | 77 |
| **Embase** | | |
| 1 | exp schizophrenia/ | 179193 |
| 2 | exp ‘schizoaffective psychosis’/ | 11784 |
| 3 | schizophreni:ab,ti | 169650 |
| 4 | OR schizoaffective:ab,ti | 9078 |
| 5 | 1 OR 2 OR 3 OR 4 | 213169 |
| 6 | exp 'transcranial magnetic stimulation'/ | 25242 |
| 7 | “transcranial magnetic stimulation”:ab,ti | 23689 |
| 8 | “theta burst stimulation”:ab,ti | 2583 |
| 9 | 5 OR 6 OR 7 | 31562 |
| 10 | 5 AND 9 | 1457 |
| 11 | Limited to (Article or Article in Press or Letter or Preprint) | 654 |
| 12 | Limited to Adult<18 to 64 years> or aged<65+ years> | 364 |
| **PsycINFO** | | |
| 1 | SU.exact("SCHIZOPHRENIA") | 106367 |
| 2 | TI(schizophreni* OR schizoaffective) | 73026 |
| 3 | AB(schizophreni* OR schizoaffective*) | 116,441 |
| 4 | 1 OR 2 OR 3 | 135593 |
| 5 | SU.exact("Transcranial Magnetic Stimulation") | 11040 |
| 6 | TI(“transcranial magnetic stimulation” OR “theta burst stimulation”) | 3792 |
| 7 | AB(“transcranial magnetic stimulation” OR “theta burst stimulation”) | 9508 |
| 8 | 5 OR 6 OR 7 | 12612 |
| 9 | 4 AND 8 | 644 |
|  | NOT SU.exact("Animals") | 628 |
|  | NOT stype.exact("Dissertations & Theses" OR "Books") | 607 |
|  | NOT me.exact(“Literature Review” OR "Systematic Review" OR "Meta Analysis") | 471 |
|  | Limited to Adulthood | 331 |
| **Web of Science - Science Citation Index Expanded** | | |
| 1 | TS=("Schizophrenia") | 137657 |
| 2 | TI=(schizophreni*) OR AB=(schizophreni*) | 106180 |
| 3 | TI=(schizoaffective) OR AB=(schizoaffective) | 4932 |
| 4 | 1 OR 2 OR 3 | 219014 |
| 5 | TS=("transcranial magnetic stimulation") | 19678 |
| 6 | TI=("transcranial magnetic stimulation") OR AB=(“transcranial magnetic stimulation”) | 14288 |
| 7 | TI=("theta burst stimulation") OR AB=(“theta burst stimulation”) | 1616 |
| 8 | 5 OR 6 OR 7 | 26923 |
| 9 | 4 AND 8 | 728 |
| 10 | NOT AB=(adolescen* OR babies OR baby OR boy OR boys OR boyhood OR girlhood OR child* OR girl* OR infan* OR juvenil* OR juvenile* OR kids* OR minors OR minors* OR neonat* OR neo-nat* OR newborn* OR new-born* OR paediatric* OR pediatric* OR perinat* OR preschool* OR puber* OR pubescen* OR school child* OR school* OR schoolchild* OR teen* OR toddler* OR underage* OR under-age* OR youth*) | 1134 |
| 11 | Limited to Article, Letter, Early Access | 728 |
| 12 | Exclude Proceeding Paper or Editorial Material or Review Article | 700 |

**Supplemental Table 2. Data Extracted from Included Studies**

| **Study** | **N** | **Symptom Target** | **rTMS Protocol** | **rTMS Target** | **Sessions** | **Treatment Intensity** | **Seizure** | | **Headache or Scalp Pain** | | **Neck Pain** | | **Dizziness or**  **Syncope** | | **Fatigue** | | **Cognitive Impairment** | |
| --- | --- | --- | --- | --- | --- | --- | --- | --- | --- | --- | --- | --- | --- | --- | --- | --- | --- | --- |
|  |  |  |  |  |  |  | **Active** | **Sham** | **Active** | **Sham** | **Active** | **Sham** | **Active** | **Sham** | **Active** | **Sham** | **Active** | **Sham** |
| Agrawal 2021^1^ | 5 | OCD sx | 1 Hz | L SMA | 20 | - | 0 | - | - | - | - | - | - | - | - | - | 0 |  |
| Bais 2014^2^ | 51 | AVH | 1 Hz | L TPJ or Bilateral (L then R) TPJ | 12 | 90% RMT | 0 | 0 | 9 | 2 | - | - | 1 | - | - | - | 0 | 1 |
| Barr 2012^3^ | 31 | Negative sx | 20 Hz | Bilateral DLPFC (sequential, i.e., R to L or L to R) | 20 | 90% RMT | 0 | 0 | 0 | 1 | 0 | 0 | - | 0 | 0 | 0 | 0 | 0 |
| Basavaraju 2021^4^ | 60 | Negative sx | iTBS | Cerebellar vermis area (VIIB) | 10 | 100% AMT | 0 | 0 | - | - | 1 | - | - | - | - | - | 0 | 0 |
| Bation 2021^5^ | 22 | Negative sx | iTBS | Left DLPFC (MTG, junction between Brodmann areas 9 and 46) | 20 | 80% RMT | 0 | 0 | - | - | - | - | - | - | - | - | 0 | 0 |
| Bidzinski 2022^6^ | 19 | Cannabis use and cognitive sx | 20 Hz | L and R DLPFC | 20 | 90% RMT | 0 | 0 | 6 | 3 | 1 | 0 | 1 | 0 | - | - | 0 | 0 |
| Blumberger 2012^7^ | 51 | AH |  | Heschl’s gyrus | 20 | LFL 115% RMT, Priming 90-115% RMT | 0 | 0 | 4 | 2 | - | - | - | - | - | - | 0 | 0 |
| Boden 2021^8^ | 16 | Negative sx | iTBS | DMPFC | 20 | 90% RMT | 0 | 0 | - | - | - | - | - | - | - | - | 0 | 0 |
| Boutros 2000^9^ | 7 | Encephalogram (EEG) changes | 1 Hz | Dominant posterior temporoparietal region | 4 | 80% RMT | 0 | - | - | - | - | - | - | - | - | - | - | - |
| Brady 2019^10^ | 11 | Negative sx | iTBS | Cerebellar vermis | 10 | 100% AMT | 0 | 0 | - | - | - | - | - | - | - | - | - | - |
| Brunelin 2023^11^ | 14 | AH | 1 Hz | L TPJ (T3-P3 according to 10/20 system) | 30 | 110% RMT | 0 | - | 2 | - | - | - | - | - | - | - | 0 | - |
| Chauhan 2021^12^ | 36 | General SZ sx, cognitive sx, and global improvement | iTBS | Vermal cerebellum, 1 cm below inion | 10 | 80% RMT | 0 | 0 | 5 | 2 | - | - | - | - | - | - | 0 | 0 |
| Chithra 2022^13^ | 3 | AH | cTBS | L TPJ | 25 | 120% RMT | 0 | - | 3 | - | - | - | - | - | - | - | 0 | - |
| Chung 2006^14^ | 1 | AH | 1 Hz | L TPC | 11 | 90% RMT | 0 | - | - | - | - | - | 1 | - | - | - | 0 | - |
| Chung 2007^15^ | 1 | AH | 1 Hz | L TPC | 15 | 90% RMT | 0 | - | - | - | - | - | 1 | - | - | - | 0 | - |
| Cohen 1999^16^ | 6 | Negative sx | 20 Hz | L PFC, orbital area, on the C3 and C4 EEG point | 10 | 80% MT | 0 | - | - | - | - | - | - | - | - | - | 0 | - |
| Cordes 2009^17^ | 70 | Negative sx | 10 Hz | L DLPFC | 15 | 110% RMT | 0 | 0 | - | - | - | - | - | - | - | - |  | - |
| Ćurčić-Blake 2022^18^ | 13 | Causal connectivity between DLPFC and IPL | 10 Hz | R DLPFC | 1 | 60% of maximal machine output | 0 | - | - | - | - | - | - | - | - | - | 0 | - |
| D'Alfonso 2002^19^ | 9 | AH and neurocognitive sx | 1 Hz | L auditory cortex | 10 | 80% MT | 0 | - | - | - | - | - | - | - | - | - | 0 | - |
| deJesus 2011^20^ | 17 | AH and general SZ sx | 1 Hz | L TPC | 20 | 90% MT | 0 | 0 | 2 | - | - | - | - | - | - | - | 0 | 0 |
| Demirtas-Tatlidede 2010^21^ | 8 | General SZ sx and cognitive sx | iTBS | Cerebellar vermis | 10 | 100% AMT | 0 | - | - | - | - | - | - | - | - | - | 0 | - |
| Dlabac-deLange 2015^22^ | 24 | Negative sx | 10 Hz | Bilateral DLPFC | 30 | 90% MT | 0 | 0 | - | - | - | - | - | - | - | - | 0 | 0 |
| Dlabac-deLange 2015^23^ | 32 | Negative sx | 10 Hz | Bilateral DLPFC | 30 | 90% MT | 0 | 0 | - | - | - | - | - | - | - | - | 0 | 0 |
| Dollfus 2008^24^ | 1 | AH | First round: 1 Hz Second round: 20 Hz | First round: Left TPC Second round: left superior temporal sulcus | First round: 10 Second round: 4 | First round: 90% RMT Second round: 80% RMT | 0 | - | - | - | - | - | - | - | - | - | 0 | - |
| Dollfus 2018^25^ | 74 | AVH | 20 Hz | L superior temporal sulcus | 4 | 80% RMT | 0 | 0 | - | - | - | - | - | - | - | - | 0 | 0 |
| Eberle 2010^26^ | 1 | AH | cTBS | Bilateral TPC | 45 | 80% AMT | 0 | - | 0 | - | 0 | - | 0 | - | 0 | - | 0 | - |
| Feinsod 1998^27^ | 10 | General SZ sx | 1 Hz | R PFC | 10 | 1 tesla | 0 | - | 0 | - | 0 | - | 0 | - | 0 | - | 0 | - |
| Fitzgerald 2004^28^ | 26 | Motor cortical excitability and cortical inhibition | 1 Hz | L motor cortex, targeting activity of R abductor pollicis brevis | 1 | 110% RMT | 0 | - | - | - | - | - | - | - | - | - | 0 | - |
| Fitzgerald 2005^29^ | 33 | AH | 1 Hz | L TPC | 10 | 90% RMT | 0 | 0 | - | - | - | - | - | - | - | - | 0 | 0 |
| Fitzgerald 2008^30^ | 20 | Negative sx | 10 Hz | Bilateral PFC | 15 | 110% RMT | 0 | 0 | 4 | 2 | - | - | - | - | - | - | 0 | 0 |
| Francis 2019^31^ | 20 | Cognitive sx | 20 Hz | Bilateral DLPFC | 10 | 110% RMT | 0 | 0 | 5 | 4 | - | - | - | - | - | - | 0 | 0 |
| Franck 2003^32^ | 1 | AVH | 1 Hz | L TPC | 10 | 90% of maximum power | 0 | - | 0 | - | 0 | - | 0 | - | 0 | - | 0 | - |
| Gan 2021^33^ | 40 | Negative sx | 10 Hz | Bilateral DMPFC | 20 | 100-120% RMT | 0 | 0 | 2 | 0 | - | - | - | - | - | - | 0 | 0 |
| Garg 2013^34^ | 1 | AVH | 5 Hz for first 7 trains, 6 Hz for next 7, 7 Hz for remaining 6 trains | Cerebellar vermis | 4 | 100% RMT | 0 | - | - | - | - | - | - | - | - | - | 0 | - |
| Garg 2016^35^ | 47 | Positive, negative, and depressive sx | 10 trains each at 5, 6, 7 Hz | Cerebellar vermis | 10 | 100% RMT | 0 | 0 | 5 | 0 | - | - | - | - | 1 | 0 | 0 | 0 |
| Geller 1997^36^ | 10 | General SZ sx | 0.033 Hz | Bilateral PFC | 1 | 100% stimulus intensity | 0 | - | - | - | - | - | - | - | - | - | 0 | - |
| Ghanbari Jolfaei 2016^37^ | 1 | Visual hallucinations | 1 Hz | Visual Cortex | 12 | 100% MT | 0 | - | - | - | - | - | - | - | - | - | 0 | - |
| Goyal 2007^38^ | 10 | Negative and positive sx | 10 Hz | L DLPFC | 10 | 110% RMT | 0 | 0 | 2 | - | - | - | - | - | - | - | 0 | 0 |
| Goyal 2015^39^ | 1 | AH | 1 Hz | L TPC | 10 | 90% RMT | 0 | - | - | - | - | - | - | - | - | - | 0 | - |
| Gupta 2021^40^ | 40 | Memory sx | 1 Hz | L TPC | 10 | 100% RMT | 0 | - | - | - | - | - | - | - | - | - | 0 | - |
| Hajak 2004^41^ | 20 | Positive, negative, and depressive sx | 10 Hz | L DLPFC | 10 | 110% MT | 0 | 0 | 3 | - | - | - | - | - | - | - | - | 0 |
| Hallmayer 2005^42^ | 39 | AH | 1 Hz | L or R TPC | 10 | 100% RMT | 0 | 0 | 5 | 2 | - | - | 2 | 1 | - | - | 1 | 1 |
| Hoffman 1999^43^ | 3 | AH | 1 Hz | L TPC | 4 | 80% RMT | 0 | 0 | - | - | - | - | - | - | - | - | 0 | 0 |
| Hoffman 2000^44^ | 12 | AH | 1 Hz | L TPC | 4 | 80% RMT | 0 | 0 | 2 | 0 | 0 | 0 | 0 | 0 | 0 | 0 | 0 | 0 |
| Hoffman 2003^45^ | 24 | AH | 1 Hz | L TPC | 9 | 90% MT | 0 | 0 | 6 | 1 | - | - | 4 | 1 | - | - | 4 | 3 |
| Hoffman 2005^46^ | 50 | AH | 1 Hz | L TPC | 9 | 90% RMT | 0 | 0 | - | - | - | - | - | - | - | - | 3 | 1 |
| Hoffman 2013^47^ | 83 | AH | 1 Hz | Wernicke's area and its homologue | 15 | 90% RMT | 0 | 0 | 1 | 0 | - | - | - | - | - | - | 2 | 0 |
| Holi 2004^48^ | 22 | General SZ sx | 10 Hz | L DLPFC | 10 | 100% MT | 0 | 0 | 8 | 0 | - | - | - | - | - | - | 0 | 0 |
| Horacek 2007^49^ | 12 | LORETA, PET, and AH | 0.9 Hz | L TPC | 10 | 100% RMT | 0 | - | - | - | - | - | - | - | - | - | 0 |  |
| Huang 2016^50^ | 41 | Cigarette smoking | 10 Hz | L DLPFC | 21 | 110% MT | 0 | 0 | 2 | 1 | - | - | - | - | - | - | 0 | 0 |
| Jandl 2005^51^ | 10 | Negative sx and electroencephalogram (EEG) | 10 Hz | L DLPFC | 5 | 100% RMT | 0 | - | - | - | - | - | - | - | - | - | 0 | - |
| Jandl 2006^52^ | 16 | AH | 1 Hz | L TPC, R TPC | 5 | 100% RMT | 0 | 0 | 1 | - | - | - | - | - | - | - | 0 | 0 |
| Jin 2006^53^ | 27 | Negative sx | 3 Hz, alpha (8-13 Hz), 20 Hz | Bilateral DLPFC | 10 | 80% RMT | 0 | 0 | - | - | - | - | - | - | - | - | 0 | 0 |
| Kar 2016^54^ | 1 | AH | 1 Hz | L TPC, R TPC | 20 | 100% RMT | 0 | - | - | - | - | - | - | - | - | - | 0 |  |
| Kim 2014^55^ | 23 | AH | 1 Hz, 20 Hz | Temporoparietal area or Broca's area | 6 (20Hz TP, 20 Hz Broca's) 10 (1 Hz TP) | 100% RMT | 0 | 0 | 20 | 5 | - | - | 6 | 0 | - | - | 0 | 0 |
| Kindler 2013^56^ | 30 | AVH | 1 Hz, iTBS (30 Hz bursts separated by 100 msec) | L TPC | 10 | 90% MT | 0 | - | 4 | - | - | - | - | - | - | - | 0 | - |
| Kindler 2013^57^ | 24 | AVH | 1 Hz, cTBS | L TPC | 10 | 90% RMT | 0 | - | 6 |  | - | - |  |  | - | - | 0 | - |
| Klein 1999^58^ | 35 | Positive, negative, and depressive sx | 1 Hz | R PFC | 10 | 110% RMT | 0 | 0 | 2 | - | - | - | - | - | - | - | 0 | 0 |
| Klirova 2013^59^ | 15 | AH | 0.9 Hz | L TPC | 10 | 100% MT | 0 | 0 | - | - | - | - | - | - | - | - | 0 | 0 |
| Koops 2016^60^ | 71 | AVH | iTBS (50 Hz burst every 200ms) | L TPC | 10 | 80% RMT | 0 | 0 | 17 | 15 | - | - | 20 | 12 | 5 | 5 | 0 | 0 |
| Kozak 2018^61^ | 13 | Smoking behavior and cognitive sx | 20 Hz | Bilateral DLPFC | 6 |  | 0 | 0 | - | - | - | - | - | - | - | - | 0 | 0 |
| Kumar 2020^62^ | 100 | Negative sx | 20 Hz | L DLPFC | 20 | 100% MT | 1 | 0 | - | - | - | - | - | - | - | - | 0 | 0 |
| Lee 2005^63^ | 39 | AH | 1 Hz | L or R TPC | 10 | 100% RMT | 0 | 0 | 5 | 2 | - | - | 2 | 1 | - | - | 1 | 1 |
| Levkovitz 2011^64^ | 15 | Negative and cognitive sx | 20 Hz | Bilateral PFC | 20 | 120% RMT | 1 | - | - | - | - | - | - | - | - | - | 0 | - |
| Li 2020^65^ | 250 | Negative and cognitive sx | 10 Hz | L DLPFC | 10 | 80% MT | 0 | - | - | - | - | - | - | - | - | - | 0 | - |
| Linsambarth 2019^66^ | 16 | Negative sx | 18 Hz | Bilateral PFC | 24 | 120% MT | 0 | - | - | - | - | - | - | - | - | - | 0 | - |
| Loo 2010^67^ | 18 | AH | 1 Hz | L or R STG | 3 | 110% RMT | 0 | 0 | - | - | - | - | - | - | - | - | 0 | 0 |
| McIntosh 2004^68^ | 16 | AH | 1 Hz | L TPC | 4 | 80% RMT | - | - | - | - | - | - | - | - | - | - | - | - |
| Mendes-Filho 2016^69^ | 12 | OCD sx | 1 Hz | SMA | 20 | 100% RMT | 0 | 0 | - | - | - | - | - | - | - | - | 0 | 0 |
| Mittrach 2010^70^ | 35 | Cognitive sx | 10 Hz | L DLPFC | 10 | 110% RMT | 0 | 0 | - | - | - | - | - | - | - | - | 0 | 0 |
| Moeller 2022^71^ | 20 | Smoking behavior | 10 Hz | Bilateral insula, VLPFC, & DLPFC | 15 | 120% RMT | 0 | 0 | 5 | - | - | 1 | - | - | - | - | 0 | 0 |
| Mogg 2007^72^ | 17 | Negative sx | 10 Hz | L DLPFC | 10 | 110% MT | 0 | 0 | - | - | - | - | - | - | - | - | 0 | 0 |
| Montagne-Larmurier 2009^73^ | 11 | AH | 20 Hz | Posterior part of left STG | 4 | 80% RMT | 0 |  | 2 | - | - | -- | - | - | - | - | 0 | - |
| Novak 2006^74^ | 18 | Negative sx | 20 Hz | L DLPFC | 10 | 90% MT | 0 | 0 | 1 | 0 | - | - | - | - | - | - | 0 | 0 |
| Oh 2011^75^ | 10 | Positive, negative, and depressive sx | L DLPFC: 10 Hz; L TPC: 1 Hz | L DLPFC and L TPC | 15 | 80-100% MT | 0 | - | - | - | - | - | - | - | - | - | 0 | - |
| Oxley 2004^76^ | 12 | Primary motor cortical excitability, cortical inhibition, and resting motor threshold | 1 Hz | L Premotor Cortex | 1 | 90% AMT | 0 | - | - | - | - | - | - | - | - | - | 0 | - |
| Paillere-Martinot 2017^77^ | 27 | AH | 1 Hz | fMRI Peak Activation in Language Recognition Task (STG n=8, MTG n=7) | 10 | 100% MT | 0 | 0 | 5 | 1 | - | - | - | - | - | - | 0 | 0 |
| Plewnia 2014^78^ | 16 | AH | cTBS | Bilateral TPC | 15 | 80% RMT | 0 | 0 | 1 | - | - | - | - | - | - | - | 0 | 0 |
| Poulet 2005^79^ | 10 | AH | 1 Hz | L TPC | 10 | 90% MT | 0 | 0 | 0 | 1 | - | - | - | - | - | - | 0 | 0 |
| Poulet 2009^80^ | 1 | AH | 1st Course: 1Hz; 2nd Course: cTBS | L TPC | 1st Course: 10; 2nd Course: 90 | 1st Course: 100% MT; 2nd Course: 80% MT | 0 | - | - | - | - | - | - | - | - | - | 0 | - |
| Prikryl 2007^81^ | 22 | Negative sx | 10 Hz | L DLPFC | 15 | 110% MT | 0 | 0 | 2 | - | - | - | - | - | - | - | 0 | 0 |
| Prikryl 2010^82^ | 1 | Positive and negative sx and cortical inhibition | L DLPFC: 10 Hz; L TPC: 0.9 Hz | L DLPFC & L TPC | 15 | 110% MT | 0 | 0 | - | - | - | - | - | - | - | - | 0 | 0 |
| Prikryl 2011^83^ | 1 | Psychosis sx | 10 Hz | L DLPFC | 1 | 110% MT | 0 | - | - | - | - | - | - | - | - | - | 0 | - |
| Prikryl 2013^84^ | 45 | Negative sx | 10 Hz | L DLPFC | 15 | 110% MT | 0 | 0 | - | - | - | - | - | - | - | - | 0 | 0 |
| Prikryl 2014^85^ | 40 | Cigarette consumption | 10 Hz | L DLPFC | 15 | 110% MT | 0 | 0 | 1 | - | - | - | - | - | - | - | 0 | 0 |
| Quan 2015^86^ | 117 | Negative sx | 10 Hz | L DLPFC, 5 cm anterior to point of maximum abductor pollicis brevis stimulation | 20, 10 per 2 week course | 80% | 0 | 0 | 3 | 1 | 0 | 0 | 0 | 0 | 0 | 0 | 0 | 0 |
| Rachid 2013^87^ | 1 | AH | 1st Course: 1 Hz; 2nd Course: cTBS | Left TPC | 1st Course: 20; 2nd Course: 20, then maintenance weekly to biweekly | First Course:130% MT 2nd Course: 80% RMT | 0 | - | - | - | - | - | - | - | - | - | 0 | - |
| Rollnik 2000^88^ | 12 | General psychiatric, anxiety, and depressive sx | 20 Hz | DLPFC of dominant hemisphere | 10 | 80% MT | 0 | 0 | 3 | 0 | - | - | - | - | - | - | 0 | 0 |
| Rollnik 2001^89^ | 1 | General psychiatric, anxiety, and depressive sx | 20 Hz | L PFC | 20 | 80% MT | 0 | 0 | - | - | - | - | - | - | - | - | 0 | 0 |
| Rosa 2007^90^ | 11 | AH | 1 Hz | L TPC | 10 | 90% MT | 0 | 0 | 1 | - | - | - | - | - | - | - | 0 | 0 |
| Rosenberg 2011^91^ | 8 | AH | 1 Hz | L TPC | 10 (62.5%) or 20 (37.5%) | 110% MT | 0 |  | 1 | - | - | - | - | - | - | - | 0 | - |
| Saba 2006^92^ | 18 | Psychosis sx | 1 Hz | L TPC | 10 | 80% MT | 0 | 0 | 2 | - | - | - | - | - | - | - | 0 | 0 |
| Sachdev 2005^93^ | 4 | Negative sx | 15 Hz | L DLPFC | 20 | 90% MT | 0 | - | - | - | - | - | - | - | - | - | 0 | - |
| Schonfeldt-Lecuona 2004^94^ | 12 | AH | 1 Hz | L STG; Broca's | 5 | 90% MT | 0 | 0 | - | - | - | - | - | - | - | - | 0 | 0 |
| Sidhoumi 2010^95^ | 1 | AH, cognitive sx, and neuronal excitability | cTBS | L TPC | 20 | 80% MT | 0 | - | - | - | - | - | - | - | - | - | 0 | - |
| Singh 2020^96^ | 30 | Negative sx | 20 Hz | L DLPFC | 20 | 100% MT | 1 | 0 | 8 | 8 | - | - | - | - | - | - | 0 | 0 |
| Slotema 2011^97^ | 62 | AVH | 1 Hz | Peak fMRI Activation during AVH: n=20; L TPC: n=22 | 15 | 90% MT | 0 | 0 | 9 | 0 | 1 | 0 | 1 | 1 | 1 | 0 | 0 | 0 |
| Slotema 2012^98^ | 23 | AVH | Priming: 6 Hz x 5 min, then 1 Hz x 15 min; No Priming: 1 Hz x 20 min | Left TPC | 15 | Priming: 80% RMT, then 90% RMT; No Priming: 90% RMT | 0 | - | 4 | - | 1 | - | - | - | - | - | 0 | - |
| Sommer 2007^99^ | 15 | AVH | 1 Hz | fMRI Peak Activation during AVH; L TPC | 15 | 90% MT | - | - | - | - | - | - | - | - | - | - | - | - |
| Stanford 2011^100^ | 5 | Negative sx | 20 Hz | L DLPFC | 20 | 100% of MT | 0 | - | 0 | - | 0 | - | - | - | - | - | 0 | - |
| Su 2022^101^ | 109 | Neurocognitive sx | "High Frequency" | L DLPFC | 20 | 110% MT | 0 | 0 | - | - | - | - | - | - | - | - | 0 | 0 |
| Su 2022^102^ | 47 | Positive and negative sx | 10 Hz | L DLPFC | 20 | 110% MT | 0 | 0 | 7 | 1 | - | - | 1 | 1 | - | - | 0 | 0 |
| Su 2022^103^ | 47 | Weight loss | 10 Hz | L DLPFC | 20 | 110% MT | 0 | 0 | 0 | 2 | - | - | 1 | 1 | - | - | 0 | 0 |
| Subramanian 2010^104^ | 1 | AH | 1 Hz | L TPC, then Bilateral TPC | 22 | 100% MT | 0 | - | - | - | - | - | - | - | - | - | 0 | - |
| Subramanian 2013^105^ | 4 | AH | 1 Hz | L TPC | 10 | - | 0 | - | 1 | - | - | - | - | - | - | - | 0 | - |
| Sverak 2014^106^ | 1 | Negative and positive sx | 10 Hz | L DLPFC | 16 | 110% RMT | 0 | - | 1 | - | - | - | - | - | - | - | 0 | - |
| Sverak 2022^107^ | 19 | Negative sx | 10 Hz | L DLPFC | 16 | 110% RMT | 0 | - | 12 | - | - | - | - | - | - | - | 0 | - |
| Thirthalli 2008^108^ | 1 | AH | 1 Hz | L TPC | 22, continuing monthly | 100% MT | 0 | - | 1 | - | - | - | - | - | - | - | 0 | - |
| Tikka 2017^109^ | 20 | First-rank sx | cTBS | R Inferior Parietal Lobule | 10 | 80% RMT | 0 | 0 | 4 | 4 | - | - | - | - | - | - | 0 | 0 |
| Tyagi 2022^110^ | 59 | AVH | cTBS | Bilatera TPC | 20 | 80% RMT | 0 | 0 | 5 | 4 | - | - | - | - | - | - | 0 | 0 |
| Van Lutterveld 2012^111^ | 32 | AH | 1 Hz | L TPC, R TPC, or Centro-occipital cortex | 1 | 90% MT | 0 | - | - | - | - | - | - | - | - | - | 0 | - |
| Vercammen 2009^112^ | 38 | AVH | 1 HZ | L TPC, Bilateral TPC | 12 | 90% MT | 0 | 0 | 8 | 1 | - | - | 1 | - | - | - | 0 | 0 |
| Voineskos 2021^113^ | 83 | Working memory and brain structure | 20 Hz | Bilateral DLPFC | 20 | 90% RMT | 0 | 0 | 33 | 16 | - | - | 1 | 0 | - | - | 0 | 0 |
| Wagner 2019^114^ | 26 | Negative sx | 10 Hz | L DLPFC | 15 | 110% RMT | 0 | 0 | - | - | - | - | - | - | - | - | 0 | 0 |
| Walther 2020^115^ | 20 | Hand gesture deficits | iTBS, cTBS | iTBS: L inferior frontal gyrus; cTBS: R inferior parietal lobe | 1 | iTBS: 80% RMT; cTBS: 100% RMT | 0 | 0 | 3 | 2 | 3 | 2 | - | - | 5 | 4 | 0 | 0 |
| Wang 2022^116^ | 59 | Visual-spatial working memory | iTBS | L DLPFC | 42 | 80% RMT | 0 | 0 | - | - | - | - | - | - | - | - | 0 | 0 |
| Wen 2021^117^ | 52 | Negative sx and cognitive sx | 10 Hz | L DLPFC | 20 | 110% MT | 0 | 0 | 2 | 2 | - | - | - | - | - | - | 0 | 0 |
| Wing 2012^118^ | 15 | Tobacco craving | 20 Hz | Bilateral DLPFC | 20 | 90% RMT | 0 | 0 | - | - | - | - | - | - | - | - | 0 | 0 |
| Wobrock 2015^119^ | 197 | Negative sx | 10 Hz | L DLPFC | 15 | 110% RMT | 0 | 0 | 14 | 4 | 0 | 0 | 0 | 0 | 1 | 1 | 0 | 0 |
| Xiu 2020^120^ | 120 | Negative sx and cognitive sx | 10 Hz; 20 Hz | L DLPFC | 40 | 110% MT | 0 | 0 | 4 | 0 | - | - | 3 | 2 | - | - | 0 | 0 |
| Yu 2002^121^ | 5 | Positive, negative, and cognitive sx | 10 Hz | L DLPFC | 5 | - | 0 | - | - | - | - | - | - | - | - | - | 0 | - |
| Zeeuws 2010^122^ | 1 | General SZ sx and psychomotor activity | 20 Hz | L DLPFC | 20 | 120% MT | 0 | - | - | - | - | - | - | - | - | - | 0 | - |
| Zhao 2014^123^ | 96 | Negative sx | 10 Hz, 20 Hz, iTBS | L DLPFC | 20 | 10 Hz & 20 Hz: 80-110% MT; iTBS: 80% MT | 0 | 0 | 1 | 1 | - | - | - | - | - | - | 0 | 0 |
| Zhu 2021^124^ | 64 | Negative sx | iTBS | Midline cerebellum | 10 | 100% RMT | 0 | 0 | 3 | - | - | - | 3 | - | - | - | 0 | 0 |
| Zhuo 2019^125^ | 70 | Negative sx and cognitive sx | 20 Hz | L DLPFC | 20 | 90% RMT | 0 | 0 | 4 | 3 | 0 | 0 | 1 | 0 | 0 | 0 | 0 | 0 |
| Zhuo 2022^126^ | 450 | Cognitive sx | 10 Hz | Occipital Lobe; Frontal Lobe (both conditions +/- Li) | 72 | 110% MT | 0 | - | 23 | - | - | - | 64 | - | - | - | 0 | - |

“-“ indicates that the data is not available; AH: auditory hallucinations; AMT: active motor threshold; AVH: auditory verbal hallucinations; cTBS: continuous theta burst stimulation; DLPFC: dorsolateral prefrontal cortex; DMPFC: dorsomedial prefrontal cortex; iTBS: intermittent theta burst stimulation; L: left; MTG: middle temporal gyrus; MT: motor threshold; R: right; RMT: resting motor threshold; OCD: obsessive compulsive disorder; PFC: prefrontal cortex; SMA: supplementary motor area; STG: superior temporal gyrus; Sx: symptoms; SZ: schizophrenia; TPC: temporoparietal cortex; TPJ: temporoparietal junction; VLPFC: ventrolateral prefrontal cortex

**Supplemental Table 2. Data Extracted from Included Studies (Continued)**

| **Study** | **N** | **Symptom Target** | **rTMS Protocol** | **rTMS Target** | **Sessions** | **Treatment Intensity** | **Worsening Psychosis** | | **Worsening Depression** | | **Worsening Mania** | | **Facial Twitching** | | **Nausea** | | **Insomnia** | |
| --- | --- | --- | --- | --- | --- | --- | --- | --- | --- | --- | --- | --- | --- | --- | --- | --- | --- | --- |
|  |  |  |  |  |  |  | **Active** | **Sham** | **Active** | **Sham** | **Active** | **Sham** | **Active** | **Sham** | **Active** | **Sham** | **Active** | **Sham** |
| Agrawal 2021^1^ | 5 | OCD sx | 1 Hz | L SMA | 20 | - | 0 | - | 0 | - | 0 | - | - | - | - | - | - | - |
| Bais 2014^2^ | 51 | AVH | 1 Hz | L TPJ or Bilateral (L then R) TPJ | 12 | 90% RMT | 0 | 0 | 0 | 0 | 0 | - | 10 | 1 | - | - | - | - |
| Barr 2012^3^ | 31 | Negative sx | 20 Hz | Bilateral DLPFC (sequential, i.e., R to L or L to R) | 20 | 90% RMT | 0 | 0 | 0 | 0 | 0 | 0 | - | - | - | - | - | - |
| Basavaraju 2021^4^ | 60 | Negative sx | iTBS | Cerebellar vermis area (VIIB) | 10 | 100% AMT | - | 0 | 0 | 0 | 2 | 0 | - | - | - | - | - | - |
| Bation 2021^5^ | 22 | Negative sx | iTBS | Left DLPFC (MTG, junction between Brodmann areas 9 and 46) | 20 | 80% RMT | 0 | 2 | 0 | 0 | 0 | 0 | - | - | - | - | - | - |
| Bidzinski 2022^6^ | 19 | Cannabis use and cognitive sx | 20 Hz | L and R DLPFC | 20 | 90% RMT | 0 | 0 | 0 | 0 | 0 | 0 | - | - | - | - | - | - |
| Blumberger 2012^7^ | 51 | AH |  | Heschl’s gyrus | 20 | LFL 115% RMT, Priming 90-115% RMT | 0 | 0 | 0 | 0 | 0 | 0 | 4 | - | - | - | - | - |
| Boden 2021^8^ | 16 | Negative sx | iTBS | DMPFC | 20 | 90% RMT | 0 | 0 | 0 | 0 | 0 | 0 | - | - | - | - | - | - |
| Boutros 2000^9^ | 7 | Encephalogram (EEG) changes | 1 Hz | Dominant posterior temporoparietal region | 4 | 80% RMT | - | - | - | - | - | - | - | - | - | - | - | - |
| Brady 2019^10^ | 11 | Negative sx | iTBS | Cerebellar Vermis | 10 | 100% AMT | - | - | - | - | - | - | - | - | - | - | - | - |
| Brunelin 2023^11^ | 14 | AH | 1 Hz | Left TPJ (T3-P3 according to 10/20 system) | 30 | 110% RMT | 0 | - | 0 | - | 0 | - | - | - | - | - | - | - |
| Chauhan 2021^12^ | 36 | General SZ sx, cognitive sx, and global improvement | iTBS | Vermal cerebellum, 1 cm below inion | 10 | 80% RMT | 0 | 0 | 0 | 0 | 0 | 0 | - | - | - | - | - | 3 |
| Chithra 2022^13^ | 3 | AH | cTBS | L TPJ | 25 | 120% RMT | 0 | - | 0 | - | 0 | - | - | - | - | - | - | - |
| Chung 2006^14^ | 1 | AH | 1 Hz | L TPC | 11 | 90% RMT | 0 | - | 0 | - | 0 | - | - | - | - | - | - | - |
| Chung 2007^15^ | 1 | AH | 1 Hz | L TPC | 15 | 90% RMT | 0 | - | 0 | - | 0 | - | - | - | - | - | - | - |
| Cohen 1999^16^ | 6 | Negative sx | 20 Hz | L PFC, orbital area, on the C3 and C4 EEG point | 10 | 80% MT | 0 | - | 0 | - | 0 | - | - | - | - | - | - | - |
| Cordes 2009^17^ | 70 | Negative sx | 10 Hz | L DLPFC | 15 | 110% RMT | - | - | - | - | - | - | - | - | - | - | - | - |
| Ćurčić-Blake 2022^18^ | 13 | Causal connectivity between DLPFC and IPL | 10 Hz | R DLPFC | 1 | 60% of maximal machine output | 0 | - | 0 | - | 0 | - | - | - | - | - | - | - |
| D'Alfonso 2002^19^ | 9 | AH and neurocognitive sx | 1 Hz | L auditory cortex | 10 | 80% MT | 0 | - | 0 | - | 0 | - | - | - | - | - | - | - |
| deJesus 2011^20^ | 17 | AH and general SZ sx | 1 Hz | L TPC | 20 | 90% MT | 0 | 0 | 0 | 0 | 0 | 0 | - | - | - | - | - | - |
| Demirtas-Tatlidede 2010^21^ | 8 | General SZ sx and cognitive sx | iTBS | Cerebellar vermis | 10 | 100% AMT | 0 | - | 0 | - | 0 | - | - | - | - | - | - | - |
| Dlabac-deLange 2015^22^ | 24 | Negative sx | 10 Hz | Bilateral DLPFC | 30 | 90% MT | 0 | 0 | 0 | 0 | 0 | 0 | - | - | - | - | - | - |
| Dlabac-deLange 2015^23^ | 32 | Negative sx | 10 Hz | Bilateral DLPFC | 30 | 90% MT | 0 | 0 | 0 | 0 | 0 | 0 | - | - | - | - | - | - |
| Dollfus 2008^24^ | 1 | AH | First round: 1 Hz Second round: 20 Hz | First round: Left TPC Second round: left superior temporal sulcus | First round: 10 Second round: 4 | First round: 90% RMT Second round: 80% RMT | 0 | - | 0 | - | 0 | - | - | - | - | - | - | - |
| Dollfus 2018^25^ | 74 | AVH | 20 Hz | L superior temporal sulcus | 4 | 80% RMT | 0 | 0 | 0 | 0 | 0 | 0 | - | - | - | - | - | - |
| Eberle 2010^26^ | 1 | AH | cTBS | Bilateral TPC | 45 | 80% AMT | 0 | - | 0 | - | 0 | - | - | - | - | - | - | - |
| Feinsod 1998^27^ | 10 | General SZ sx | 1 Hz | R PFC | 10 | 1 tesla | 0 | - | 0 | - | 0 | - | - | - | - | - | - | - |
| Fitzgerald 2004^28^ | 26 | Motor cortical excitability and cortical inhibition | 1 Hz | L motor cortex, targeting activity of R abductor pollicis brevis | 1 | 110% RMT | 0 | - | 0 | - | 0 | - | - | - | - | - | - | - |
| Fitzgerald 2005^29^ | 33 | AH | 1 Hz | L TPC | 10 | 90% RMT | 0 | - | 0 | - | 0 | - | - | - | - | - | - | - |
| Fitzgerald 2008^30^ | 20 | Negative sx | 10 Hz | Bilateral PFC | 15 | 110% RMT | 0 | 0 | 0 | 0 | 0 | 0 | - | - | - | - | - | - |
| Francis 2019^31^ | 20 | Cognitive sx | 20 Hz | Bilateral DLPFC | 10 | 110% RMT | 0 | 0 | 0 | 0 | 0 | 0 | - | 1 | - | - | - | - |
| Franck 2003^32^ | 1 | AVH | 1 Hz | L TPC | 10 | 90% of maximum power | 0 | - | 0 | - | 0 | - | 1 | - | - | - | - | - |
| Gan 2021^33^ | 40 | Negative sx | 10 Hz | Bilateral DMPFC | 20 | 100-120% RMT | 0 | 0 | 0 | 0 | 0 | 0 | - | - | - | - | - | - |
| Garg 2013^34^ | 1 | AVH | 5 Hz for first 7 trains, 6 Hz for next 7, 7 Hz for remaining 6 trains | Cerebellar vermis | 4 | 100% RMT | 1 | - | 0 | - | 0 | - | - | - | - | - | - | - |
| Garg 2016^35^ | 47 | Positive, negative, and depressive sx | 10 trains each at 5, 6, 7 Hz | Cerebellar vermis | 10 | 100% RMT | 0 | 0 | 0 | 0 | 0 | 0 | - | - | - | - | - | - |
| Geller 1997^36^ | 10 | General SZ sx | 0.033 Hz | Bilateral PFC | 1 | 100% stimulus intensity | 0 | - | 0 | - | 0 | - | - | - | - | - | - | - |
| Ghanbari Jolfaei 2016^37^ | 1 | Visual hallucinations | 1 Hz | Visual cortex | 12 | 100% MT | 0 | - | 0 | - | 0 | - | - | - | - | - | - | - |
| Goyal 2007^38^ | 10 | Negative and positive sx | 10 Hz | L DLPFC | 10 | 110% RMT | 0 | 0 | 0 | 0 | 0 | 0 | - | - | - | - | - | - |
| Goyal 2015^39^ | 1 | AH | 1 Hz | L TPC | 10 | 90% RMT | 0 | - | 0 | - | 0 | - | - | - | - | - | - | - |
| Gupta 2021^40^ | 40 | Memory sx | 1 Hz | L TPC | 10 | 100% RMT | 0 | - | 0 | - | 0 | - | - | - | - | - | - | - |
| Hajak 2004^41^ | 20 | Positive, negative, and depressive sx | 10 Hz | L DLPFC | 10 | 110% MT | 0 | 0 | 0 | 0 | 0 | 0 | - | - | - | - | - | - |
| Hallmayer 2005^42^ | 39 | AH | 1 Hz | L or R TPC | 10 | 100% RMT | 0 | 0 | 0 | 0 | 0 | 0 | - | - | - | - | - | - |
| Hoffman 1999^43^ | 3 | AH | 1 Hz | L TPC | 4 | 80% RMT | 0 | 0 | 0 | 0 | 0 | 0 | - | - | - | - | - | - |
| Hoffman 2000^44^ | 12 | AH | 1 Hz | L TPC | 4 | 80% RMT | 0 | 0 | 0 | 0 | 0 | 0 | - | - | - | - | - | - |
| Hoffman 2003^45^ | 24 | AH | 1 Hz | L TPC | 9 | 90% MT | 4 | 2 | 0 | 0 | 1 | 0 | - | - | - | - | - | - |
| Hoffman 2005^46^ | 50 | AH | 1 Hz | L TPC | 9 | 90% RMT | 0 | 1 | 0 | 0 | 0 | 0 | - | - | - | - | - | - |
| Hoffman 2013^47^ | 83 | AH | 1 Hz | Wernicke's area and R homologue | 15 | 90% RMT | 0 | 3 | 0 | 0 | 0 | 0 | - | - | - | - | - | - |
| Holi 2004^48^ | 22 | General SZ sx | 10 Hz | L DLPFC | 10 | 100% MT | 0 | 0 | 0 | 0 | 0 | 0 | - | - | - | - | - | - |
| Horacek 2007^49^ | 12 | LORETA, PET, and AH | 0.9 Hz | L TPC | 10 | 100% RMT | 0 | - | 0 | - | 0 | - | - | - | - | - | - | - |
| Huang 2016^50^ | 41 | Cigarette smoking | 10 Hz | L DLPFC | 21 | 110% MT | 0 | 0 | 0 | 0 | 0 | 0 | - | - | - | - | - | - |
| Jandl 2005^51^ | 10 | Negative sx and electroencephalogram (EEG) | 10 Hz | L DLPFC | 5 | 100% RMT | 0 | - | 0 | - | 0 | - | - | - | - | - | - | - |
| Jandl 2006^52^ | 16 | AH | 1 Hz | L TPC, R TPC | 5 | 100% RMT | 0 | 0 | 0 | 0 | 0 | 0 | - | 1 | - | - | - | - |
| Jin 2006^53^ | 27 | Negative sx | 3 Hz, alpha (8-13 Hz), 20 Hz | Bilateral DLPFC | 10 | 80% RMT | 0 | 0 | 0 | 0 | 0 | 0 | - | - | - | - | - | - |
| Kar 2016^54^ | 1 | AH | 1 Hz | L TPC, R TPC | 20 | 100% RMT | 1 | - | 0 | - | 0 | - | - | - | - | - | - | - |
| Kim 2014^55^ | 23 | AH | 1 Hz, 20 Hz | Temporoparietal area or Broca's area | 6 (20Hz TP, 20 Hz Broca's) 10 (1 Hz TP) | 100% RMT | 0 | 0 | 0 | 0 | 0 | 0 | - | - | 0 | - | - | - |
| Kindler 2013^56^ | 30 | AVH | 1 Hz, iTBS (30 Hz bursts separated by 100 msec) | L TPC | 10 | 90% MT | 0 | - | 0 | - | 0 | - | - | - | - | - | - | - |
| Kindler 2013^57^ | 24 | AVH | 1 Hz, cTBS | L TPC | 10 | 90% RMT | 0 | - | 0 | - | 0 | - | - | - | - | - | - | - |
| Klein 1999^58^ | 35 | Positive, negative, and depressive sx | 1 Hz | R PFC | 10 | 110% RMT | 0 | 0 | 0 | 0 | 0 | 0 | 3 | - | - | - | - | - |
| Klirova 2013^59^ | 15 | AH | 0.9 Hz | L TPC | 10 | 100% MT | 0 | 0 | 0 | 0 | 0 | 0 | - | - | - | - | - | 8 |
| Koops 2016^60^ | 71 | AVH | iTBS (50 Hz burst every 200ms) | L TPC | 10 | 80% RMT | 0 | 0 | 0 | 0 | 0 | 0 | - | - | 12 | - | - | - |
| Kozak 2018^61^ | 13 | Smoking behavior and cognitive sx | 20 Hz | Bilateral DLPFC | 6 |  | 0 | 0 | 0 | 0 | 0 | 0 | - | - | - | - | - | - |
| Kumar 2020^62^ | 100 | Negative sx | 20 Hz | L DLPFC | 20 | 100% MT | 1 | 0 | 0 | 0 | 0 | 0 | - | - | - | - | - | - |
| Lee 2005^63^ | 39 | AH | 1 Hz | L or R TPC | 10 | 100% RMT | 0 | 0 | 0 | 0 | 0 | 0 | - | - | - | - | - | 1 |
| Levkovitz 2011^64^ | 15 | Negative and cognitive sx | 20 Hz | Bilateral PFC | 20 | 120% RMT | 0 | - | 0 | - | 0 | - | - | - | - | - | - | - |
| Li 2020^65^ | 250 | Negative and cognitive sx | 10 Hz | L DLPFC | 10 | 80% MT | 0 | - | 0 | - | 0 | - | - | - | - | - | - | - |
| Linsambarth 2019^66^ | 16 | Negative sx | 18 Hz | Bilateral PFC | 24 | 120% MT | 0 | - | 0 | - | 0 | - | - | - | - | - | - | - |
| Loo 2010^67^ | 18 | AH | 1 Hz | L or R STG | 3 | 110% RMT | 0 | 0 | 0 | 0 | 0 | 0 | - | 2 | - | - | - | - |
| McIntosh 2004^68^ | 16 | AH | 1 Hz | L TPC | 4 | 80% RMT | - | - | - | - | - | - | - | - | - | - | - | - |
| Mendes-Filho 2016^69^ | 12 | OCD sx | 1 Hz | SMA | 20 | 100% RMT | 0 | 0 | 0 | 0 | 0 | 0 | - | - | - | - | - | - |
| Mittrach 2010^70^ | 35 | Cognitive sx | 10 Hz | L DLPFC | 10 | 110% RMT | 0 | 0 | 0 | 0 | 0 | 0 | - | - | - | - | - | - |
| Moeller 2022^71^ | 20 | Smoking behavior | 10 Hz | Bilateral insula, VLPFC, & DLPFC | 15 | 120% RMT | 0 | 0 | 0 | 0 | 0 | 0 | 3 | - | - | - | - | - |
| Mogg 2007^72^ | 17 | Negative sx | 10 Hz | Left DLPFC | 10 | 110% MT | 0 | 0 | 0 | 0 | 0 | 0 | - | - | - | - | - | - |
| Montagne-Larmurier 2009^73^ | 11 | AH | 20 Hz | Posterior part of left Superior Temporal Sulcus | 4 | 80% RMT | 0 | - | 0 | - | 0 | - | - | - | - | - | - | - |
| Novak 2006^74^ | 18 | Negative sx | 20 Hz | L DLPFC | 10 | 90% MT | 0 | 0 | 0 | 0 | 0 | 0 | - | - | - | - | - | - |
| Oh 2011^75^ | 10 | Positive, negative, and depressive sx | L DLPFC: 10 Hz; L TPC: 1 Hz | L DLPFC and L TPC | 15 | 80-100% MT | 1 | - | 0 | - | 0 | - | - | - | - | - | - | - |
| Oxley 2004^76^ | 12 | Primary motor cortical excitability, cortical inhibition, and resting motor threshold | 1 Hz | L Premotor Cortex | 1 | 90% AMT | 0 | - | 0 | - | 0 | - | - | - | - | - | - | - |
| Paillere-Martinot 2017^77^ | 27 | AH | 1 Hz | fMRI Peak Activation in Language Recognition Task (STG n=8, MTG n=7) | 10 | 100% MT | 0 | 0 | 0 | 0 | 0 | 0 | - | - | - | - | - | - |
| Plewnia 2014^78^ | 16 | AH | cTBS | Bilateral TPC | 15 | 80% RMT | 0 | 0 | 0 | 0 | 0 | 0 | - | - | - | - | - | - |
| Poulet 2005^79^ | 10 | AH | 1 Hz | L TPC | 10 | 90% MT | 0 | 0 | 0 | 0 | 0 | 0 | - | - | - | - | - | - |
| Poulet 2009^80^ | 1 | AH | 1st Course: 1Hz; 2nd Course: cTBS | L TPC | 1st Course: 10; 2nd Course: 90 | 1st Course: 100% MT; 2nd Course: 80% MT | 0 | - | 0 | - | 0 | - | - | - | - | - | - | - |
| Prikryl 2007^81^ | 22 | Negative sx | 10 Hz | L DLPFC | 15 | 110% MT | 0 | 0 | 0 | 0 | 0 | 0 | - | - | - | - | - | - |
| Prikryl 2010^82^ | 1 | Positive and negative sx and cortical inhibition | L DLPFC: 10 Hz; L TPC: 0.9 Hz | L DLPFC & L TPC | 15 | 110% MT | 0 | 0 | 0 | 0 | 0 | 0 | - | - | - | - | - | - |
| Prikryl 2011^83^ | 1 | Psychotic sx | 10 Hz | L DLPFC | 1 | 110% MT | 1 | - | 0 | - | 0 | - | - | - | - | - | - | - |
| Prikryl 2013^84^ | 45 | Negative sx | 10 Hz | L DLPFC | 15 | 110% MT | 0 | 0 | 0 | 0 | 0 | 0 | - | - | - | - | - | - |
| Prikryl 2014^85^ | 40 | Cigarette consumption | 10 Hz | L DLPFC | 15 | 110% MT | 0 | 0 | 0 | 0 | 0 | 0 | - | - | - | - | - | - |
| Quan 2015^86^ | 117 | Negative sx | 10 Hz | L DLPFC, 5 cm anterior to point of maximum abductor pollicis brevis stimulation | 20, 10 per 2 week course | 80% | 0 | 0 | 0 | 0 | 0 | 0 | - | - | - | - | - | - |
| Rachid 2013^87^ | 1 | AH | 1st Course: 1 Hz; 2nd Course: cTBS | Left TPC | 1st Course: 20; 2nd Course: 20, then maintenance weekly to biweekly | First Course:130% MT 2nd Course: 80% RMT | 0 | - | 0 | - | 0 | - | - | - | - | - | - | - |
| Rollnik 2000^88^ | 12 | General psychiatric, anxiety, and depressive sx | 20 Hz | DLPFC of dominant hemisphere | 10 | 80% MT | 0 | 0 | 0 | 0 | 0 | 0 | - | - | - | - | - | - |
| Rollnik 2001^89^ | 1 | General psychiatric, anxiety, and depressive sx | 20 Hz | L PFC | 20 | 80% MT | 0 | 0 | 0 | 0 | 0 | 0 | - | - | - | - | - | - |
| Rosa 2007^90^ | 11 | AH | 1 Hz | L TPC | 10 | 90% MT | 0 | 0 | 0 | 0 | 0 | 0 | - | - | - | - | - | - |
| Rosenberg 2011^91^ | 8 | AH | 1 Hz | L TPC | 10 (62.5%) or 20 (37.5%) | 110% MT | 0 | - | 0 | - | 0 |  | - | - | - | - | - | - |
| Saba 2006^92^ | 18 | Psychosis sx | 1 Hz | L TPC | 10 | 80% MT | 0 | 0 | 0 | 0 | 0 | 0 | - | - | - | - | - | - |
| Sachdev 2005^93^ | 4 | Negative sx | 15 Hz | L DLPFC | 20 | 90% MT | 0 | - | 0 | - | 0 | - | - | - | - | - | - | - |
| Schonfeldt-Lecuona 2004^94^ | 12 | AH | 1 Hz | L STG; Broca's | 5 | 90% MT | 0 | 0 | 0 | 0 | 0 | 0 | - | - | - | - | - | - |
| Sidhoumi 2010^95^ | 1 | AH, cognitive sx, and neuronal excitability | cTBS | L TPC | 20 | 80% MT | 0 | - | 0 | - | 0 | - | - | - | - | - | - | - |
| Singh 2020^96^ | 30 | Negative sx | 20 Hz | L DLPFC | 20 | 100% MT | 0 | 0 | 0 | 0 | 0 | 0 | 1 | - | - | - | - | - |
| Slotema 2011^97^ | 62 | AVH | 1 Hz | Peak fMRI Activation during AVH: n=20; L TPC: n=22 | 15 | 90% MT | 2 | 3 | 0 | 0 | 0 | 0 | 7 | - | 1 | - | - | - |
| Slotema 2012^98^ | 23 | AVH | Priming: 6 Hz x 5 min, then 1 Hz x 15 min; No Priming: 1 Hz x 20 min | L TPC | 15 | Priming: 80% RMT, then 90% RMT; No Priming: 90% RMT | 0 | - | 0 | - | 0 | - | 2 | - | - | - | - | - |
| Sommer 2007^99^ | 15 | AVH | 1 Hz | fMRI Peak Activation during AVH; L TPC | 15 | 90% MT | 2 | - |  | - |  | - | - | - | - | - | - | - |
| Stanford 2011^100^ | 5 | Negative sx | 20 Hz | L DLPFC | 20 | 100% of MT | 0 | - | 0 | - | 0 | - | - | - | - | - | - | - |
| Su 2022^101^ | 109 | Neurocognitive sx | "High Frequency" | L DLPFC | 20 | 110% MT | 0 | 0 | 0 | 0 | 0 | 0 | - | - | - | - | - | - |
| Su 2022^102^ | 47 | Positive and negative sx | 10 Hz | L DLPFC | 20 | 110% MT | 0 | 0 | 0 | 0 | 0 | 0 | - | - | - | - | - | - |
| Su 2022^103^ | 47 | Weight loss | 10 Hz | L DLPFC | 20 | 110% MT | 0 | 0 | 0 | 0 | 0 | 0 | - | - | - | - | - | - |
| Subramanian 2010^104^ | 1 | AH | 1 Hz | Left TPC, then Bilateral TPC | 22 | 100% MT | 1 | - | 0 | - | 0 | - | - | - | - | - | - | - |
| Subramanian 2013^105^ | 4 | AH | 1 Hz | L TPC | 10 |  | 0 | - | 0 | - | 0 | - | - | - | - | - | - | - |
| Sverak 2014^106^ | 1 | Negative and positive sx | 10 Hz | L DLPFC | 16 | 110% RMT | 0 | - | 0 | - | 0 | - | - | - | - | - | - | - |
| Sverak 2022^107^ | 19 | Negative sx | 10 Hz | L DLPFC | 16 | 110% RMT | 0 | - | 0 | - | 0 | - | - | - | - | 1 | 1 | - |
| Thirthalli 2008^108^ | 1 | AH | 1 Hz | L TPC | 22, continuing monthly | 100% MT | 0 | - | 0 | - | 0 | - | - | - | - | - | - | - |
| Tikka 2017^109^ | 20 | First-rank sx | cTBS | R Inferior Parietal Lobule | 10 | 80% RMT | 1 | 1 | 0 | 0 | 0 | 0 | - | - | - | - | - | - |
| Tyagi 2022^110^ | 59 | AVH | cTBS | Bilateral TPC | 20 | 80% RMT | 0 | 0 | 0 | 0 | 0 | 0 | - | - | - | - | - | - |
| Van Lutterveld 2012^111^ | 32 | AH | 1 Hz | L TPC, R TPC, or Centro-occipital cortex | 1 | 90% MT | 0 | - | 0 | - | 0 | - | - | - | - | - | - | - |
| Vercammen 2009^112^ | 38 | AVH | 1 HZ | L TPC, Bilateral TPC | 12 | 90% MT | 1 | 0 | 0 | 0 | 0 | 0 | 7 | 1 | - | - | - | - |
| Voineskos 2021^113^ | 83 | Working memory and brain structure | 20 Hz | Bilateral DLPFC | 20 | 90% RMT | 2 | 0 | 0 | 2 | 0 | 0 | - | - | 1 | 1 | 1 | - |
| Wagner 2019^114^ | 26 | Negative sx | 10 Hz | L DLPFC | 15 | 110% RMT | 1 | 0 | 0 | 0 | 0 | 0 | - | - | - | - | - | - |
| Walther 2020^115^ | 20 | Hand gesture deficits | iTBS, cTBS | iTBS: L inferior frontal gyrus; cTBS: R inferior parietal lobe | 1 | iTBS: 80% RMT; cTBS: 100% RMT | 0 | 0 | 0 | 0 | 0 | 0 | - | - | 5 | - | - | - |
| Wang 2022^116^ | 59 | Visual-spatial working memory | iTBS | L DLPFC | 42 | 80% RMT | 0 | 0 | 0 | 0 | 0 | 0 | - | - | - | 5 | 5 | - |
| Wen 2021^117^ | 52 | Negative sx and cognitive sx | 10 Hz | L DLPFC | 20 | 110% MT | 0 | 0 | 0 | 0 | 0 | 0 | - | - | - | - | - | - |
| Wing 2012^118^ | 15 | Tobacco craving | 20 Hz | Bilateral DLPFC | 20 | 90% RMT | 0 | 0 | 0 | 0 | 0 | 0 | - | - | - | - | - | - |
| Wobrock 2015^119^ | 197 | Negative sx | 10 Hz | L DLPFC | 15 | 110% RMT | 2 | 1 | 1 | 1 | 0 | 0 | 3 | - | - | - | - | - |
| Xiu 2020^120^ | 120 | Negative sx and cognitive sx | 10 Hz; 20 Hz | L DLPFC | 40 | 110% MT | 0 | 0 | 0 | 0 | 0 | 0 | - | - | - | - | - | - |
| Yu 2002^121^ | 5 | Positive, negative, and cognitive sx | 10 Hz | L DLPFC | 5 |  | 0 | - | 0 | - | 0 | - | - | - | - | - | - | - |
| Zeeuws 2010^122^ | 1 | General SZ sx and psychomotor activity | 20 Hz | L DLPFC | 20 | 120% MT | 0 | - | 0 | - | 0 | - | - | - | - | - | - | - |
| Zhao 2014^123^ | 96 | Negative sx | 10 Hz, 20 Hz, iTBS | L DLPFC | 20 | 10 Hz & 20 Hz: 80-110% MT; iTBS: 80% MT | 0 | 0 | 0 | 0 | 0 | 0 | - | - | - | - | - | - |
| Zhu 2021^124^ | 64 | Negative sx | iTBS | Midline cerebellum | 10 | 100% RMT | 0 | 0 | 0 | 0 | 0 | 0 | - | - | - | - | - | - |
| Zhuo 2019^125^ | 70 | Negative sx and cognitive sx | 20 Hz | L DLPFC | 20 | 90% RMT | 0 | 0 | 0 | 0 | 0 | 0 | - | - | - | - | - | - |
| Zhuo 2022^126^ | 450 | Cognitive sx | 10 Hz | Occipital Lobe; Frontal Lobe (both conditions +/- Li) | 72 | 110% MT | 0 | - | 0 | - | 0 | - | - | - | 54 | - | - | - |

*“-” indicates that the data is not available; AH: auditory hallucinations; AMT: active motor threshold; AVH: auditory verbal hallucinations; cTBS: continuous theta burst stimulation; DLPFC: dorsolateral prefrontal cortex; DMPFC: dorsomedial prefrontal cortex; iTBS: intermittent theta burst stimulation; L: left; MTG: middle temporal gyrus; MT: motor threshold; R: right; RMT: resting motor threshold; OCD: obsessive compulsive disorder; PFC: prefrontal cortex; SMA: supplementary motor area; STG: superior temporal gyrus; Sx: symptoms; SZ: schizophrenia; TPC: temporoparietal cortex; TPJ: temporoparietal junction; VLPFC: ventrolateral prefrontal cortex

**Supplemental Table 3. Quality Assessment of Included Studies**^*^

| **Study** | **Sequence generation** | **Allocation concealment** | **Blinding of participants and personnel** | **Blinding of outcome assessment** | **Incomplete outcome data** | **Selective reporting** | **Other sources of bias** |
| --- | --- | --- | --- | --- | --- | --- | --- |
| Agrawal 2021^1^ | Low | High | High | High | Low | Low | Low |
| Bais 2014^2^ | Low | Low | Low | Low | Low | Low | Low |
| Barr 2012^3^ | Low | Low | High | Low | Low | Low | Low |
| Basavaraju 2021^4^ | Low | Low | Low | Low | Low | Low | Low |
| Bation 2021^5^ | Low | Low | Low | Low | Low | Low | Low |
| Bidzinski 2022^6^ | Low | Low | Low | Low | Low | Low | Low |
| Blumberger 2012^7^ | Unsure | Unsure | Low | Low | Low | Low | Low |
| Boden 2021^8^ | Low | Low | Low | Low | Low | Low | Low |
| Boutros 2000^9^ | High | High | High | High | Low | Low | Low |
| Brady 2019^10^ | Low | Low | Low | Low | Low | Low | Low |
| Brunelin 2023^11^ | High | High | High | High | Low | Low | Low |
| Chauhan 2021^12^ | Low | Low | Low | Low | Low | Low | Low |
| Chithra 2022^13^ | High | High | High | High | Low | Low | Low |
| Chung 2006^14^ | High | High | High | High | Low | Low | Low |
| Chung 2007^15^ | High | High | High | High | Low | Low | Low |
| Cohen 1999^16^ | High | High | High | High | Low | Low | Low |
| Cordes 2009^17^ | Low | Low | Low | Low | Low | Low | Low |
| Ćurčić-Blake 2022^18^ | Low | High | High | High | Low | Low | Low |
| D'Alfonso 2002^19^ | High | High | High | High | Low | Low | Low |
| deJesus 2011^20^ | Low | Low | Low | Low | Unsure | Low | Low |
| Demirtas-Tatlidede 2010^21^ | Low | High | High | High | Low | Low | Low |
| Dlabac-deLange 2015^22^ | Low | Low | Low | Low | Low | Low | Low |
| Dlabac-deLange 2015^23^ | Low | Low | Low | Low | Low | Low | Low |
| Dollfus 2008^24^ | High | High | High | High | Low | Low | Low |
| Dollfus 2018^25^ | Low | Low | Low | Low | Low | Low | Low |
| Eberle 2010^26^ | High | High | High | High | Low | High | Low |
| Feinsod 1998^27^ | High | High | High | High | Low | Low | Low |
| Fitzgerald 2004^28^ | High | High | High | High | Low | Low | Low |
| Fitzgerald 2005^29^ | Low | Low | Low | Unsure | Low | Low | Low |
| Fitzgerald 2008^30^ | Low | Low | Low | Low | Low | Low | Low |
| Francis 2019^31^ | Low | Low | Low | Low | Low | Low | Low |
| Franck 2003^32^ | High | High | High | High | Low | Low | Low |
| Gan 2021^33^ | Low | Low | Low | Low | Low | Low | Low |
| Garg 2013^34^ | High | High | High | High | Low | Low | Low |
| Garg 2016^35^ | Low | Low | Low | Low | Low | Low | Low |
| Geller 1997^36^ | High | High | High | High | Low | Low | Low |
| Ghanbari Jolfaei 2016^37^ | High | High | High | High | Low | Low | Low |
| Goyal 2007^38^ | Low | Low | Low | Low | Low | Low | Low |
| Goyal 2015^39^ | High | High | High | Low | Low | Low | Low |
| Gupta 2021^40^ | High | High | High | High | Low | Unsure | High |
| Hajak 2004^41^ | Low | Low | Low | Low | Low | Low | Low |
| Hallmayer 2005^42^ | Low | Low | Low | Low | Low | Low | Low |
| Hoffman 1999^43^ | Low | Low | Low | Low | Low | Low | High |
| Hoffman 2000^44^ | Low | Low | Low | Low | Low | Low | Low |
| Hoffman 2003^45^ | Low | Low | Low | Low | Low | Low | Low |
| Hoffman 2005^46^ | Low | Low | Low | Low | Low | Low | Low |
| Hoffman 2013^47^ | Low | Low | Low | Low | Low | Low | Low |
| Holi 2004^48^ | Low | Low | Low | Low | Low | Low | Low |
| Horacek 2007^49^ | High | High | High | Low | Low | Low | Low |
| Huang 2016^50^ | Low | Low | Low | Low | Low | Low | Low |
| Jandl 2005^51^ | High | High | High | High | Low | Low | Low |
| Jandl 2006^52^ | Low | Low | Low | Low | Low | Low | Low |
| Jin 2006^53^ | Low | Low | Low | Low | Low | Low | Low |
| Kar 2016^54^ | High | High | High | High | Low | Low | Low |
| Kim 2014^55^ | Low | Low | Low | Low | Low | Low | Low |
| Kindler 2013^56^ | Low | High | High | High | Low | Low | Low |
| Kindler 2013^57^ | Low | Low | Low | Low | Low | Low | Low |
| Klein 1999^58^ | Low | Low | Low | Low | Low | Low | Low |
| Klirova 2013^59^ | Low | Low | Low | Low | Low | Low | Low |
| Koops 2016^60^ | Low | Low | Low | Low | Low | Low | Low |
| Kozak 2018^61^ | Unsure | Unsure | Low | Unsure | Low | Low | Low |
| Kumar 2020^62^ | Low | Low | Low | Low | Low | Low | Low |
| Lee 2005^63^ | Low | Low | Low | Low | Low | Low | Low |
| Levkovitz 2011^64^ | High | High | High | High | Low | Low | Low |
| Li 2020^65^ | Low | Low | Unsure | Unsure | Low | Low | Low |
| Linsambarth 2019^66^ | High | High | High | High | Low | Low | Low |
| Loo 2010^67^ | Low | Low | Low | Low | Low | Low | Low |
| McIntosh 2004^68^ | Low | Low | Unsure | Unsure | Low | Low | Low |
| Mendes-Filho 2016^69^ | Low | Low | Low | Low | Low | Low | Low |
| Mittrach 2010^70^ | Low | Low | Low | Low | Low | Low | Low |
| Moeller 2022^71^ | Low | Low | Low | Low | Low | Low | Low |
| Mogg 2007^72^ | Low | Low | Low | Low | Low | Low | Low |
| Montagne-Larmurier 2009^73^ | High | High | High | High | Low | Low | Low |
| Novak 2006^74^ | Low | Low | Low | Low | Low | Unsure | Low |
| Oh 2011^75^ | High | High | High | High | Low | Low | Low |
| Oxley 2004^76^ | High | High | High | High | Low | Unsure | Low |
| Paillere-Martinot 2017^77^ | Low | Low | Low | Low | Low | Low | Low |
| Plewnia 2014^78^ | Low | Low | Low | Low | Low | Low | Low |
| Poulet 2005^79^ | Low | Low | Low | Low | Low | Low | Low |
| Poulet 2009^80^ | High | High | High | High | Low | Low | Low |
| Prikryl 2007^81^ | Low | Low | Low | Low | Low | Low | Low |
| Prikryl 2010^82^ | High | Low | Low | Unsure | Low | Unsure | Low |
| Prikryl 2011^83^ | High | High | High | High | Low | Low | Low |
| Prikryl 2013^84^ | Low |  | Low | Low | Low | Low | Low |
| Prikryl 2014^85^ | Low | Low | Low | Low | Low | Low | High |
| Quan 2015^86^ | Low | Low | Low | Low | Low | Low | Low |
| Rachid 2013^87^ | High | High | High | High | Low | Low | Low |
| Rollnik 2000^88^ | Low | Low | Low | Low | Low | Low | Low |
| Rollnik 2001^89^ | High | High | High | High | Low | Low | Low |
| Rosa 2007^90^ | Low | Low | Low | Low | Low | Low | Low |
| Rosenberg 2011^91^ | High | High | High | High | Low | Low | Low |
| Saba 2006^92^ | Low | Low | Low | Low | Low | Low | Low |
| Sachdev 2005^93^ | High | High | High | High | Low | Low | Low |
| Schonfeldt-Lecuona 2004^94^ | Low | Low | Low | Low | Low | Low | Low |
| Sidhoumi 2010^95^ | High | High | High | High | Low | Low | Low |
| Singh 2020^96^ | Low | Low | Low | Low | Low | Low | Low |
| Slotema 2011^97^ | Low | Low | Low | Low | Low | Low | Low |
| Slotema 2012^98^ | Low | Low | Low | Low | Low | Low | Low |
| Sommer 2007^99^ | High | High | High | High | Low | Low | Low |
| Stanford 2011^100^ | High | High | High | High | Low | Low | Low |
| Su 2022^101^ | Low | Low | Low | Low | Low | Low | High |
| Su 2022^102^ | Low | Low | Low | Low | Low | Low | Low |
| Su 2022^103^ | Low | Low | Low | Low | Low | Low | Low |
| Subramanian 2010^104^ | High | High | High | High | Low | Low | Low |
| Subramanian 2013^105^ | High | High | High | High | Low | Low | Low |
| Sverak 2014^106^ | High | High | High | High | Low | Low | Low |
| Sverak 2022^107^ | High | High | High | High | Low | Low | Low |
| Thirthalli 2008^108^ | High | High | High | High | Low | Low | Low |
| Tikka 2017^109^ | Low | Low | Low | Low | Low | Low | Low |
| Tyagi 2022^110^ | Low | Low | Low | Low | Low | Low | Low |
| Van Lutterveld 2012^111^ | High | High | Low | High | Low | Low | Low |
| Vercammen 2009^112^ | Low | Low | Low | Low | Low | Low | Low |
| Voineskos 2021^113^ | Low | Low | Low | Low | Low | Low | Low |
| Wagner 2019^114^ | Low | Low | Low | Low | Low | Low | Low |
| Walther 2020^115^ | Low | Low | Low | Low | Low | Low | Low |
| Wang 2022^116^ | Low | Low | Low | Low | Low | Low | Low |
| Wen 2021^117^ | Low | Low | Low | Low | Low | Low | Low |
| Wing 2012^118^ | Low | Low | Low | Low | Low | Low | Low |
| Wobrock 2015^119^ | Low | Low | Low | Low | Low | Low | Low |
| Xiu 2020^120^ | Low | Low | Low | Low | Low | Low | Low |
| Yu 2002^121^ | High | High | High | High | Low | Low | Low |
| Zeeuws 2010^122^ | High | High | High | High | High | High | Low |
| Zhao 2014^123^ | Low | Low | Low | Low | Low | Low | Low |
| Zhu 2021^124^ | Low | Low | Low | Low | Low | Low | High |
| Zhuo 2019^125^ | Unsure | Unsure | High | Unsure | Low | Low | Low |
| Zhuo 2022^126^ | Unsure | Unsure | High | Unsure | Unsure | High | High |

^*^Quality Assessment was performed according to the Cochrane Handbook by assessing risk of bias for each assigned category. In the table, “Low” indicates low risk of bias, whereas “High” indicates high risk of bias.

**Supplemental Table 4. Estimated Prevalence of Side Effects in TMS Targeted to Temporal/TPC**

| **Side Effect​** | **Active rTMS ​(Estimate, 95% CI)** | **All Sham rTMS ​(Estimate, 95% CI)** | **p​** |
| --- | --- | --- | --- |
| Seizure​ | 0.00/1000​ (0.00/1000-4.79/1000) | 0.00/1000​ (0.00/1000-2.71/1000) | 1 |
| Headache or Scalp Pain | 147.1/1000 (122.8/1000-174.2/1000) | 68.4/1000​ (55.5/1000-83.1/1000) | <0.0001* |
| Neck Pain​ | 5.21/1000 (1.421/1000-13.3/1000) | 2.21/1000​ (0.455/1000-6.43/1000) | 0.261 |
| Dizziness or Syncope​ | 43.0/1000 (29.8/1000-59.8/1000) | 14.7/1000 (9.01/1000-22.6/1000)​ | 0.000107* |
| Fatigue​ | 9.11/1000 (3.67/1000-18.7/1000) | 7.35/1000 (3.53/1000-13.5/1000) | 0.853 |
| Cognitive Impairment​ | 14.3/1000 (7.17/1000-25.5/1000) | 5.15/1000​ (2.07/1000-10.6/1000) | 0.0485* |
| Worsening Psychosis​ | 15.6/1000 (8.10/1000-27.1/1000) | 9.56/1000​ (5.10/1000-16.3/1000) | 0.299 |
| Worsening Depression​ | 0/1000 (0.00/1000-4.79/1000) | 2.21/1000​ (0.455/1000-6.43/1000) | 0.558 |
| Worsening Mania​ | 1.30/1000 (0.0330/1000-7.23/1000) | 0.00/1000​ (0.00/1000-2.71/1000) | 0.361 |
| Facial Twitching​ | 40.4/1000 (27.6/1000-56.8/1000) | 8.82/1000​ (4.57/1000-15.4/1000) | <0.0001* |
| Nausea​ | 19.5/1000 (11.0/1000-32.0/1000) | 5.15/1000​ (2.07/1000-10.6/1000) | 0.00342* |
| Insomnia​ | 0.00/1000 (0.00/1000-4.79/1000) | 0.735/1000 ​(0.0186/1000-4.10/1000) | 1 |

**Supplemental Table 4. Estimated Prevalence of Side Effects in TMS Targeted to Temporal/TPC**. 768 participants across 54 studies received active TMS targeted to the Temporal/TPC area.

**Supplemental Table 5. Estimated Prevalence of Side Effects in TMS Targeted to DLPFC**

| **Side Effect​** | **Active rTMS ​(Estimate, 95% CI)** | **All Sham rTMS ​(Estimate, 95% CI)** | **p​** |
| --- | --- | --- | --- |
| Seizure​ | 1.85/1000 (0.224/1000-6.67/1000) | 0.00/1000​ (0.00/1000-2.71/1000) | 0.196 |
| Headache or Scalp Pain | 112.9/1000 (94.6/1000-133.2/1000) | 68.4/1000​ (55.5/1000-83.1/1000) | 0.000162* |
| Neck Pain​ | 0.925/1000 (0.0234/1000-5.14/1000) | 2.21/1000​ (0.455/1000-6.43/1000) | 0.634 |
| Dizziness or Syncope​ | 7.40/1000 (3.20/1000-14.5/1000) | 14.7/1000 (9.01/1000-22.6/1000)​ | 0.124 |
| Fatigue​ | 0.925/1000 (0.0234/1000-5.14/1000) | 7.35/1000 (3.53/1000-13.5/1000) | 0.0284* |
| Cognitive Impairment​ | 0.00/1000 (0.00/1000-3.41/1000) | 5.15/1000​ (2.07/1000-10.6/1000) | 0.0198* |
| Worsening Psychosis​ | 6.48/1000 (2.61/1000-13.3/1000) | 9.56/1000​ (5.10/1000-16.3/1000) | 0.500 |
| Worsening Depression​ | 0.925/1000 (0.0234/1000-5.14/1000) | 2.21/1000​ (0.455/1000-6.43/1000) | 0.634 |
| Worsening Mania​ | 0.00/1000 (0.00/1000-0.341/1000) | 0.00/1000​ (0.00/1000-2.71/1000) | 1 |
| Facial Twitching​ | 3.70/1000 (1.01/1000-9.45/1000) | 8.82/1000​ (4.57/1000-15.4/1000) | 0.136 |
| Nausea​ | 0.925/1000 (0.0234/1000-5.14/1000) | 5.15/1000​ (2.07/1000-10.6/1000) | 0.0841 |
| Insomnia​ | 6.48/1000 (2.61/1000-13.3/1000) | 0.735/1000 ​(0.0186/1000-4.10/1000) | 0.0255* |

**Supplemental Table 5. Estimated Prevalence of Side Effects in TMS Targeted to DLPFC**. 1081 participants across 45 studies received active TMS targeted to the DLPFC.

**Supplemental Table 6. Estimated Prevalence of Side Effects in TMS Targeted to PFC**

| **Side Effect​** | **Active rTMS ​(Estimate, 95% CI)** | **All Sham rTMS ​(Estimate, 95% CI)** | **p​** |
| --- | --- | --- | --- |
| Seizure​ | 11.4/1000 (0.288/1000-61.7/1000) | 0.00/1000​ (0.00/1000-2.71/1000) | 0.0608 |
| Headache or Scalp Pain | 68.2/1000 (25.4/1000-142.5/1000) | 68.4/1000​ (55.5/1000-83.1/1000) | 1 |
| Neck Pain​ | 0.00/1000 (0/1000-41.1/1000) | 2.21/1000​ (0.455/1000-6.43/1000) | 1 |
| Dizziness or Syncope​ | 0.00/1000 (0.00/1000-41.1/1000) | 14.7/1000 (9.01/1000-22.6/1000)​ | 0.629 |
| Fatigue​ | 0.00/1000 (0.00/1000-41.1/1000) | 7.35/1000 (3.53/1000-13.5/1000) | 1 |
| Cognitive Impairment​ | 0.00/1000 (0.00/1000-41.1/1000) | 5.15/1000​ (2.07/1000-10.6/1000) | 1 |
| Worsening Psychosis​ | 0.00/1000 (0.00/1000-41.1/1000) | 9.56/1000​ (5.10/1000-16.3/1000) | 1 |
| Worsening Depression​ | 0.00/1000 (0.00/1000-41.1/1000) | 2.21/1000​ (0.455/1000-6.43/1000) | 1 |
| Worsening Mania​ | 0.00/1000 (0.00/1000-41.1/1000) | 0.00/1000​ (0.00/1000-2.71/1000) | 1 |
| Facial Twitching​ | 34.1/1000 (7.09/1000-96.4/1000) | 8.82/1000​ (4.57/1000-15.4/1000) | 0.0580 |
| Nausea​ | 0.00/1000 (0.00/1000-41.1/1000) | 5.15/1000​ (2.07/1000-10.6/1000) | 1 |
| Insomnia​ | 0.736/1000 (0.0186/1000-4.10/1000) | 0.735/1000 ​(0.0186/1000-4.10/1000) | 1 |

**Supplemental Table 6. Estimated Prevalence of Side Effects in TMS Targeted to PFC**. 88 participants across 8 studies received active TMS targeted to the PFC.

**Supplemental Table 7. Estimated Prevalence of Side Effects in TMS Targeted to Cerebellar Vermis**

| **Side Effect​** | **Active rTMS ​(Estimate, 95% CI)** | **All Sham rTMS ​(Estimate, 95% CI)** | **p​** |
| --- | --- | --- | --- |
| Seizure​ | 0.00/1000 (0.00/1000-30.8/1000) | 0.00/1000​ (0.00/1000-2.71/1000) | 1 |
| Headache or Scalp Pain | 100.2/1000 (60.0/1000-181.0/1000) | 68.4/1000​ (55.5/1000-83.1/1000) | 0.949 |
| Neck Pain​ | 8.47/1000 (0.215/1000-46.3/1000) | 2.21/1000​ (0.455/1000-6.43/1000) | 0.283 |
| Dizziness or Syncope​ | 25.4/1000 (5.27/1000-72.5/1000) | 14.7/1000 (9.01/1000-22.6/1000)​ | 0.422 |
| Fatigue​ | 8.47/1000 (0.215/1000-46.3/1000) | 7.35/1000 (3.53/1000-13.5/1000) | 0.601 |
| Cognitive Impairment​ | 0.00/1000 (0.00/1000-30.8/1000) | 5.15/1000​ (2.07/1000-10.6/1000) | 1 |
| Worsening Psychosis​ | 8.47/1000 (0.215/1000-46.3/1000) | 9.56/1000​ (5.10/1000-16.3/1000) | 1 |
| Worsening Depression​ | 0.00/1000 (0.00/1000-30.8/1000) | 2.21/1000​ (0.455/1000-6.43/1000) | 1 |
| Worsening Mania​ | 16.9/1000 (2.06/1000-59.9/1000) | 0.00/1000​ (0.00/1000-2.71/1000) | 0.00632* |
| Facial Twitching​ | 0.00/1000 (0.00/1000-30.8/1000) | 8.82/1000​ (4.57/1000-15.4/1000) | 0.615 |
| Nausea​ | 0.00/1000 (0.00/1000-30.8/1000) | 5.15/1000​ (2.07/1000-10.6/1000) | 1 |
| Insomnia​ | 0.00/1000 (0.00/1000-30.8/1000) | 0.735/1000 ​(0.0186/1000-4.10/1000) | 1 |

**Supplemental Table 7. Estimated Prevalence of Side Effects in TMS Targeted to Cerebellar Vermis**. 118 participants across 7 studies received active TMS targeted to the cerebellar vermis.

**Supplemental Table 8. Estimated Prevalence of Side Effects in TMS Targeted to Motor/SMA**

| **Side Effect​** | **Active rTMS ​(Estimate, 95% CI)** | **All Sham rTMS ​(Estimate, 95% CI)** | **p​** |
| --- | --- | --- | --- |
| Seizure​ | 0.00/1000 (0.00/1000-72.5/1000) | 0.00/1000​ (0.00/1000-2.71/1000) | 1 |
| Headache or Scalp Pain | 0.00/1000 (0.00/1000-72.5/1000) | 68.4/1000​ (55.5/1000-83.1/1000) | 0.0712 |
| Neck Pain​ | 0.00/1000 (0.00/1000-72.5/1000) | 2.21/1000​ (0.455/1000-6.43/1000) | 1 |
| Dizziness or Syncope​ | 0.00/1000 (0.00/1000-72.5/1000) | 14.7/1000 (9.01/1000-22.6/1000)​ | 1 |
| Fatigue​ | 0.00/1000 (0.00/1000-72.5/1000) | 7.35/1000 (3.53/1000-13.5/1000) | 1 |
| Cognitive Impairment​ | 0.00/1000 (0.00/1000-72.5/1000) | 5.15/1000​ (2.07/1000-10.6/1000) | 1 |
| Worsening Psychosis​ | 0.00/1000 (0.00/1000-72.5/1000) | 9.56/1000​ (5.10/1000-16.3/1000) | 1 |
| Worsening Depression​ | 0.00/1000 (0.00/1000-72.5/1000) | 2.21/1000​ (0.455/1000-6.43/1000) | 1 |
| Worsening Mania​ | 0.00/1000 (0.00/1000-72.5/1000) | 0.00/1000​ (0.00/1000-2.71/1000) | 1 |
| Facial Twitching​ | 0.00/1000 (0.00/1000-72.5/1000) | 8.82/1000​ (4.57/1000-15.4/1000) | 1 |
| Nausea​ | 0.00/1000 (0.00/1000-72.5/1000) | 5.15/1000​ (2.07/1000-10.6/1000) | 1 |
| Insomnia​ | 0.00/1000 (0.00/1000-72.5/1000) | 0.735/1000 ​(0.0186/1000-4.10/1000) | 1 |

**Supplemental Table 8. Estimated Prevalence of Side Effects in TMS Targeted to Motor/SMA**. 49 participants across 4 studies received active TMS targeted to the Motor/SMA region.

**Supplemental Table 9. Estimated Prevalence of Side Effects in TMS Targeted to VLPFC**

| **Side Effect​** | **Active rTMS ​(Estimate, 95% CI)** | **All Sham rTMS ​(Estimate, 95% CI)** | **p​** |
| --- | --- | --- | --- |
| Seizure​ | 0.00/1000 (0.00/1000-123.4/1000) | 0.00/1000​ (0.00/1000-2.71/1000) | 1 |
| Headache or Scalp Pain | 35.7/1000 (0.904/1000-183.5/1000) | 68.4/1000​ (55.5/1000-83.1/1000) | 1 |
| Neck Pain​ | 35.7/1000 (0.904/1000-183.5/1000) | 2.21/1000​ (0.455/1000-6.43/1000) | 0.0784 |
| Dizziness or Syncope​ | 0.00/1000 (0.00/1000-123.4/1000) | 14.7/1000 (9.01/1000-22.6/1000)​ | 1 |
| Fatigue​ | 142.9/1000 (40.3/1000-326.7/1000) | 7.35/1000 (3.53/1000-13.5/1000) | 0.000116* |
| Cognitive Impairment​ | 0.00/1000 (0.00/1000-123.4/1000) | 5.15/1000​ (2.07/1000-10.6/1000) | 1 |
| Worsening Psychosis​ | 0.00/1000 (0.00/1000-123.4/1000) | 9.56/1000​ (5.10/1000-16.3/1000) | 1 |
| Worsening Depression​ | 0.00/1000 (0.00/1000-123.4/1000) | 2.21/1000​ (0.455/1000-6.43/1000) | 1 |
| Worsening Mania​ | 0.00/1000 (0.00/1000-123.4/1000) | 0.00/1000​ (0.00/1000-2.71/1000) | 1 |
| Facial Twitching​ | 0.00/1000 (0.00/1000-123.4/1000) | 8.82/1000​ (4.57/1000-15.4/1000) | 1 |
| Nausea​ | 107.1/1000 (22.7/1000-282.3/1000) | 5.15/1000​ (2.07/1000-10.6/1000) | 0.000804* |
| Insomnia​ | 0.00/1000 (0.00/1000-123.4/1000) | 0.735/1000 ​(0.0186/1000-4.10/1000) | 1 |

**Supplemental Table 9. Estimated Prevalence of Side Effects in TMS Targeted to VLPFC**. 28 participants across two studies received active TMS targeted to the VLPFC region.

**Supplemental Table 10. Estimated Prevalence of Side Effects in TMS Targeted to DMPFC**

| **Side Effect​** | **Active rTMS ​(Estimate, 95% CI)** | **All Sham rTMS ​(Estimate, 95% CI)** | **p​** |
| --- | --- | --- | --- |
| Seizure​ | 0.00/1000 (0.00/1000-132.3/1000) | 0.00/1000​ (0.00/1000-2.71/1000) | 1 |
| Headache or Scalp Pain | 76.9/1000 (9.46/1000-251.3/1000) | 68.4/1000​ (55.5/1000-83.1/1000) | 0.697 |
| Neck Pain​ | 0.00/1000 (0.00/1000-132.3/1000) | 2.21/1000​ (0.455/1000-6.43/1000) | 1 |
| Dizziness or Syncope​ | 0.00/1000 (0.00/1000-132.3/1000) | 14.7/1000 (9.01/1000-22.6/1000)​ | 1 |
| Fatigue​ | 0.00/1000 (0.00/1000-132.3/1000) | 7.35/1000 (3.53/1000-13.5/1000) | 1 |
| Cognitive Impairment​ | 0.00/1000 (0.00/1000-132.3/1000) | 5.15/1000​ (2.07/1000-10.6/1000) | 1 |
| Worsening Psychosis​ | 0.00/1000 (0.00/1000-132.3/1000) | 9.56/1000​ (5.10/1000-16.3/1000) | 1 |
| Worsening Depression​ | 0.00/1000 (0.00/1000-132.3/1000) | 2.21/1000​ (0.455/1000-6.43/1000) | 1 |
| Worsening Mania​ | 0.00/1000 (0.00/1000-132.3/1000) | 0.00/1000​ (0.00/1000-2.71/1000) | 1 |
| Facial Twitching​ | 0.00/1000 (0.00/1000-132.3/1000) | 8.82/1000​ (4.57/1000-15.4/1000) | 1 |
| Nausea​ | 0.00/1000 (0.00/1000-132.3/1000) | 5.15/1000​ (2.07/1000-10.6/1000) | 1 |
| Insomnia​ | 0.00/1000 (0.00/1000-132.3/1000) | 0.735/1000 ​(0.0186/1000-4.10/1000) | 1 |

**Supplemental Table 10. Estimated Prevalence of Side Effects in TMS Targeted to DMPFC**. 26 participants across two studies received active TMS to the DMPFC region.

**Supplemental Table 11. Estimated Prevalence of Side Effects in TMS Targeted to Occipital**

| **Side Effect​** | **Active rTMS ​(Estimate, 95% CI)** | **All Sham rTMS ​(Estimate, 95% CI)** | **p​** |
| --- | --- | --- | --- |
| Seizure​ | 0.00/1000 (0.00/1000-975/1000) | 0.00/1000​ (0.00/1000-2.71/1000) | 1 |
| Headache or Scalp Pain | 0.00/1000 (0.00/1000-975/1000) | 68.4/1000​ (55.5/1000-83.1/1000) | 1 |
| Neck Pain​ | 0.00/1000 (0.00/1000-975/1000) | 2.21/1000​ (0.455/1000-6.43/1000) | 1 |
| Dizziness or Syncope​ | 0.00/1000 (0.00/1000-975/1000) | 14.7/1000 (9.01/1000-22.6/1000)​ | 1 |
| Fatigue​ | 0.00/1000 (0.00/1000-975/1000) | 7.35/1000 (3.53/1000-13.5/1000) | 1 |
| Cognitive Impairment​ | 0.00/1000 (0.00/1000-975/1000) | 5.15/1000​ (2.07/1000-10.6/1000) | 1 |
| Worsening Psychosis​ | 0.00/1000 (0.00/1000-975/1000) | 9.56/1000​ (5.10/1000-16.3/1000) | 1 |
| Worsening Depression​ | 0.00/1000 (0.00/1000-975/1000) | 2.21/1000​ (0.455/1000-6.43/1000) | 1 |
| Worsening Mania​ | 0.00/1000 (0.00/1000-975/1000) | 0.00/1000​ (0.00/1000-2.71/1000) | 1 |
| Facial Twitching​ | 0.00/1000 (0.00/1000-975/1000) | 8.82/1000​ (4.57/1000-15.4/1000) | 1 |
| Nausea​ | 0.00/1000 (0.00/1000-975/1000) | 5.15/1000​ (2.07/1000-10.6/1000) | 1 |
| Insomnia​ | 0.00/1000 (0.00/1000-975/1000) | 0.735/1000 ​(0.0186/1000-4.10/1000) | 1 |

**Supplemental Table 11. Estimated Prevalence of Side Effects in TMS Targeted to Occipital**. One participant in one study received active TMS to the Occipital region.

**Supplemental Table 12. Estimated Prevalence of Side Effects in TMS Targeted to Insula**

| **Side Effect​** | **Active rTMS ​(Estimate, 95% CI)** | **All Sham rTMS ​(Estimate, 95% CI)** | **p​** |
| --- | --- | --- | --- |
| Seizure​ | 0.00/1000 (0.00/1000-308.5/1000) | 0.00/1000​ (0.00/1000-2.71/1000) | 1 |
| Headache or Scalp Pain | 500/1000 (187.1/1000-812.9/1000) | 68.4/1000​ (55.5/1000-83.1/1000) | 0.000320* |
| Neck Pain​ | 0.00/1000 (0.00/1000-308.5/1000) | 2.21/1000​ (0.455/1000-6.43/1000) | 1 |
| Dizziness or Syncope​ | 0.00/1000 (0.00/1000-308.5/1000) | 14.7/1000 (9.01/1000-22.6/1000)​ | 1 |
| Fatigue​ | 0.00/1000 (0.00/1000-308.5/1000) | 7.35/1000 (3.53/1000-13.5/1000) | 1 |
| Cognitive Impairment​ | 0.00/1000 (0.00/1000-308.5/1000) | 5.15/1000​ (2.07/1000-10.6/1000) | 1 |
| Worsening Psychosis​ | 0.00/1000 (0.00/1000-308.5/1000) | 9.56/1000​ (5.10/1000-16.3/1000) | 1 |
| Worsening Depression​ | 0.00/1000 (0.00/1000-308.5/1000) | 2.21/1000​ (0.455/1000-6.43/1000) | 1 |
| Worsening Mania​ | 0.00/1000 (0.00/1000-308.5/1000) | 0.00/1000​ (0.00/1000-2.71/1000) | 1 |
| Facial Twitching​ | 300/1000 (66.7/1000-652.5/1000) | 8.82/1000​ (4.57/1000-15.4/1000) | 0.000122* |
| Nausea​ | 0.00/1000 (0.00/1000-308.5/1000) | 5.15/1000​ (2.07/1000-10.6/1000) | 1 |
| Insomnia​ | 0.00/1000 (0.00/1000-308.5/1000) | 0.735/1000 ​(0.0186/1000-4.10/1000) | 1 |

**Supplemental Table 12. Estimated Prevalence of Side Effects in TMS Targeted to Insula**. Ten participants in one study received active TMS to the Insula.

**Supplemental Table 13. Estimated Prevalence of Side Effects in High Frequency TMS**

| **Side Effect​** | **Active rTMS ​(Estimate, 95% CI)** | **All Sham rTMS ​(Estimate, 95% CI)** | **p​** |
| --- | --- | --- | --- |
| Seizure​ | 2.61/1000 (0.539/1000-7.61/1000) | 0.00/1000​ (0.00/1000-2.71/1000) | 0.0959 |
| Headache or Scalp Pain | 141.9/1000 (122.2/1000-163.4/1000) | 68.4/1000​ (55.5/1000-83.1/1000) | <0.0001* |
| Neck Pain​ | 0.870/1000 (0.0220/1000-4.84/1000) | 2.21/1000​ (0.455/1000-6.43/1000) | 0.63 |
| Dizziness or Syncope​ | 62.7/1000 (49.3/1000-78.3/1000) | 14.7/1000 (9.01/1000-22.6/1000)​ | <0.0001* |
| Fatigue​ | 1.74/1000 (0.211/1000-6.27/1000) | 7.35/1000 (3.53/1000-13.5/1000) | 0.0462* |
| Cognitive Impairment​ | 0.00/1000 (0.00/1000-3.21/1000) | 5.15/1000​ (2.07/1000-10.6/1000) | 0.0178* |
| Worsening Psychosis​ | 6.96/1000 (3.01/1000-13.7/1000) | 9.56/1000​ (5.10/1000-16.3/1000) | 0.517 |
| Worsening Depression​ | 0.870/1000 (0.0220/1000-4.84/1000) | 2.21/1000​ (0.455/1000-6.43/1000) | 0.63 |
| Worsening Mania​ | 0.00/1000 (0.00/1000-3.21/1000) | 0.00/1000​ (0.00/1000-2.71/1000) | 1 |
| Facial Twitching​ | 6.09/1000 (2.45/1000-12.5/1000) | 8.82/1000​ (4.57/1000-15.4/1000) | 0.494 |
| Nausea​ | 47.9/1000 (36.3/1000-61.9/1000) | 5.15/1000​ (2.07/1000-10.6/1000) | <0.0001* |
| Insomnia​ | 4.35/1000 (1.41/1000-10.1/1000) | 0.735/1000 ​(0.0186/1000-4.10/1000) | 0.0997 |

**Supplemental Table 13. Estimated Prevalence of Side Effects in High Frequency TMS**. 1149 participants across 56 studies received high frequency active TMS.

**Supplemental Table 14. Estimated Prevalence of Side Effects in Low Frequency TMS**

| **Side Effect​** | **Active rTMS ​(Estimate, 95% CI)** | **All Sham rTMS ​(Estimate, 95% CI)** | **p​** |
| --- | --- | --- | --- |
| Seizure​ | 0.00/1000 (0.00/1000-5.88/1000) | 0.00/1000​ (0.00/1000-2.71/1000) | 1 |
| Headache or Scalp Pain | 102.4/1000 (79.8/1000-128.9/1000) | 68.4/1000​ (55.5/1000-83.1/1000) | 0.0118* |
| Neck Pain​ | 1.60/1000 (0.0405/1000-8.88/1000) | 2.21/1000​ (0.455/1000-6.43/1000) | 1 |
| Dizziness or Syncope​ | 20.8/1000 (11.1/1000-35.3/1000) | 14.7/1000 (9.01/1000-22.6/1000)​ | 0.346 |
| Fatigue​ | 1.60/1000 (0.0405/1000-8.88/1000) | 7.35/1000 (3.53/1000-13.5/1000) | 0.190 |
| Cognitive Impairment​ | 17.6/1000 (8.82/1000-31.3/1000) | 5.15/1000​ (2.07/1000-10.6/1000) | 0.0138* |
| Worsening Psychosis​ | 17.6/1000 (8.82/1000-31.3/1000) | 9.56/1000​ (5.10/1000-16.3/1000) | 0.193 |
| Worsening Depression​ | 0.00/1000 (0.00/1000-5.88/1000) | 2.21/1000​ (0.455/1000-6.43/1000) | 0.556 |
| Worsening Mania​ | 1.60/1000 (0.0405/1000-8.88/1000) | 0.00/1000​ (0.00/1000-2.71/1000) | 0.315 |
| Facial Twitching​ | 46.4/1000 (31.3/1000-66.0/1000) | 8.82/1000​ (4.57/1000-15.4/1000) | <0.0001* |
| Nausea​ | 1.60/1000 (0.0405/1000-8.88/1000) | 5.15/1000​ (2.07/1000-10.6/1000) | 0.448 |
| Insomnia​ | 0.00/1000 (0.00/1000-5.88/1000) | 0.735/1000 ​(0.0186/1000-4.10/1000) | 1 |

**Supplemental Table 14. Estimated Prevalence of Side Effects in Low Frequency TMS**. 625 participants across 49 studies received low frequency active TMS.

**Supplemental Table 15. Estimated Prevalence of Side Effects in iTBS**

| **Side Effect​** | **Active rTMS ​(Estimate, 95% CI)** | **All Sham rTMS ​(Estimate, 95% CI)** | **p​** |
| --- | --- | --- | --- |
| Seizure​ | 0.00/1000 (0.00/1000-15.8/1000) | 0.00/1000​ (0.00/1000-2.71/1000) | 1 |
| Headache or Scalp Pain | 112.1/1000 (74.5/1000-159.9/1000) | 68.4/1000​ (55.5/1000-83.1/1000) | 0.0276* |
| Neck Pain​ | 8.62/1000 (1.05/1000-30.8/1000) | 2.21/1000​ (0.455/1000-6.43/1000) | 0.157 |
| Dizziness or Syncope​ | 99.1/1000 (63.9/1000-145.0/1000) | 14.7/1000 (9.01/1000-22.6/1000)​ | <0.0001* |
| Fatigue​ | 38.8/1000 (17.9/1000-72.4/1000) | 7.35/1000 (3.53/1000-13.5/1000) | 0.000178* |
| Cognitive Impairment​ | 0.00/1000 (0.00/1000-15.8/1000) | 5.15/1000​ (2.07/1000-10.6/1000) | 0.603 |
| Worsening Psychosis​ | 0.00/1000 (0.00/1000-15.8/1000) | 9.56/1000​ (5.10/1000-16.3/1000) | 0.236 |
| Worsening Depression​ | 0.00/1000 (0.00/1000-15.8/1000) | 2.21/1000​ (0.455/1000-6.43/1000) | 1 |
| Worsening Mania​ | 8.62/1000 (1.05/1000-30.8/1000) | 0.00/1000​ (0.00/1000-2.71/1000) | 0.0212* |
| Facial Twitching​ | 0.00/1000 (0.00/1000-15.8/1000) | 8.82/1000​ (4.57/1000-15.4/1000) | 0.234 |
| Nausea​ | 64.7/1000 (36.6/1000-104.4/1000) | 5.15/1000​ (2.07/1000-10.6/1000) | <0.0001* |
| Insomnia​ | 8.62/1000 (1.05/1000-30.8/1000) | 0.735/1000 ​(0.0186/1000-4.10/1000) | 0.0574 |

**Supplemental Table 15. Estimated Prevalence of Side Effects in iTBS**. 232 participants across 11 studies received iTBS.

**Supplemental Table 16. Estimated Prevalence of Side Effects in cTBS**

| **Side Effect​** | **Active rTMS ​(Estimate, 95% CI)** | **All Sham rTMS ​(Estimate, 95% CI)** | **p​** |
| --- | --- | --- | --- |
| Seizure​ | 0.00/1000 (0.00/1000-48.0/1000) | 0.00/1000​ (0.00/1000-2.71/1000) | 1 |
| Headache or Scalp Pain | 200/1000 (116.5/1000-308.3/1000) | 68.4/1000​ (55.5/1000-83.1/1000) | <0.0001* |
| Neck Pain​ | 26.7/1000 (3.25/1000-93.0/1000) | 2.21/1000​ (0.455/1000-6.43/1000) | 0.0243* |
| Dizziness or Syncope​ | 0.00/1000 (0.00/1000-48.0/1000) | 14.7/1000 (9.01/1000-22.6/1000)​ | 0.621 |
| Fatigue​ | 13.3/1000 (0.338/1000-72.1/1000) | 7.35/1000 (3.53/1000-13.5/1000) | 0.447 |
| Cognitive Impairment​ | 0.00/1000 (0.00/1000-48.0/1000) | 5.15/1000​ (2.07/1000-10.6/1000) | 1 |
| Worsening Psychosis​ | 13.3/1000 (0.338/1000-72.1/1000) | 9.56/1000​ (5.10/1000-16.3/1000) | 0.53 |
| Worsening Depression​ | 0.00/1000 (0.00/1000-48.0/1000) | 2.21/1000​ (0.455/1000-6.43/1000) | 1 |
| Worsening Mania​ | 0.00/1000 (0.00/1000-48.0/1000) | 0.00/1000​ (0.00/1000-2.71/1000) | 1 |
| Facial Twitching​ | 0.00/1000 (0.00/1000-48.0/1000) | 8.82/1000​ (4.57/1000-15.4/1000) | 1 |
| Nausea​ | 26.7/1000 (3.25/1000-93.0/1000) | 5.15/1000​ (2.07/1000-10.6/1000) | 0.0765 |
| Insomnia​ | 0.00/1000 (0.00/1000-48.0/1000) | 0.735/1000 ​(0.0186/1000-4.10/1000) | 1 |

**Supplemental Table 17. Estimated Prevalence of Side Effects in cTBS**. 75 participants across nine studies received cTBS.

**References**

1. Agrawal A., Joshi M., Kar S.K., Agarwal V. Role of repetitive transcranial magnetic stimulation in management of obsessive-compulsive disorder in patients of schizophrenia. *Asian J Psychiatry*. 2021;65((Agrawal, Joshi, Kar, Agarwal) King George’s Medical University, Lucknow, Uttar pradesh, India):102822. doi:10.1016/j.ajp.2021.102822

2. Bais L., Vercammen A., Stewart R., et al. Short and long term effects of left and bilateral repetitive transcranial magnetic stimulation in schizophrenia patients with auditory verbal hallucinations: A randomized controlled trial. *PLoS ONE*. 2014;9(10):e108828. doi:10.1371/journal.pone.0108828

3. Barr M.S., Farzan F., Tran L.C., Fitzgerald P.B., Daskalakis Z.J. A randomized controlled trial of sequentially bilateral prefrontal cortex repetitive transcranial magnetic stimulation in the treatment of negative symptoms in schizophrenia. *Brain Stimulat*. 2012;5(3):337-346. doi:10.1016/j.brs.2011.06.003

4. Basavaraju R., Ithal D., Thanki M.V., et al. Intermittent theta burst stimulation of cerebellar vermis enhances fronto-cerebellar resting state functional connectivity in schizophrenia with predominant negative symptoms: A randomized controlled trial. *Schizophr Res*. 2021;238((Basavaraju, Ithal, Thanki) Department of Psychiatry, National Institute of Mental Health and Neurosciences (NIMHANS), Bangalore, Karnataka, India(Ramalingaiah) Department of Neuro Imaging and Interventional Radiology, National Institute of Mental Health):108-120. doi:10.1016/j.schres.2021.10.005

5. Bation R, Magnin C, Poulet E, Mondino M, Brunelin J. Intermittent theta burst stimulation for negative symptoms of schizophrenia-A double-blind, sham-controlled pilot study. *NPJ Schizophr*. 2021;7(1):10. doi:10.1038/s41537-021-00138-3

6. Bidzinski KK, Lowe DJE, Sanches M, et al. Investigating repetitive transcranial magnetic stimulation on cannabis use and cognition in people with schizophrenia. *Schizophr Heidelb*. 2022;8(1):2. doi:10.1038/s41537-022-00210-6

7. Blumberger D.M., Christensen B.K., Zipursky R.B., et al. MRI-targeted repetitive transcranial magnetic stimulation of Heschl’s gyrus for refractory auditory hallucinations. *Brain Stimulat*. 2012;5(4):577-585. doi:10.1016/j.brs.2011.12.002

8. Boden R., Bengtsson J., Thornblom E., Struckmann W., Persson J. Dorsomedial prefrontal theta burst stimulation to treat anhedonia, avolition, and blunted affect in schizophrenia or depression - a randomized controlled trial. *J Affect Disord*. 2021;290((Boden, Bengtsson, Thornblom, Struckmann, Persson) Department of Neuroscience, Pychiatry, Uppsala University, Entrance 10, ground floor, Brain Stimulation Unit, Uppsala SE-751 85, Sweden):308-315. doi:10.1016/j.jad.2021.04.053

9. Boutros N, Berman R, Hoffman R, Miano A, Campbell D, Ilmoniemi R. Electroencephalogram and repetitive transcranial magnetic stimulation. *Depress ANXIETY*. 2000;12(3):166-169. doi:10.1002/1520-6394(2000)12:3<166::AID-DA8>3.0.CO;2-M

10. Brady R.O., Gonsalvez I., Lee I., et al. Cerebellar-prefrontal network connectivity and negative symptoms in schizophrenia. *Am J Psychiatry*. 2019;176(7):512-520. doi:10.1176/appi.ajp.2018.18040429

11. Brunelin J, Galvao F, Mondino M. Twice daily low frequency rTMS for treatment-resistant auditory hallucinations. *Int J Clin Health Psychol*. 2023;23(1):100344. doi:10.1016/j.ijchp.2022.100344

12. Chauhan P., Garg S., Tikka S.K., Khattri S. Efficacy of Intensive Cerebellar Intermittent Theta Burst Stimulation (iCiTBS) in Treatment-Resistant Schizophrenia: a Randomized Placebo-Controlled Study. *Cerebellum*. 2021;20(1):116-123. doi:10.1007/s12311-020-01193-9

13. Chithra U, Samantaray S, Kumar V, et al. Add-on accelerated continuous theta burst stimulation (a-cTBS) over the left temporoparietal junction for the management of persistent auditory hallucinations in schizophrenia: A case series. *Brain Stimul*. Published online 2022. doi:10.1016/j.brs.2022.11.005

14. Chung Y.-C., Im E.-S., Cho G.-H., Ko M.-H. Second trial of repeated transcranial magnetic stimulation in a schizophrenic patient with persistent auditory hallucinations. *Psychiatry Investig*. 2006;3(2):99-102.

15. Chung Y.-C., Im E.-S., Cho G.-H., Ko M.-H. Second run of transcranial magnetic stimulation has no effects on persistent auditory hallucinations. *World J Biol Psychiatry*. 2007;8(1):48-50. doi:10.1080/15622970600954044

16. Cohen E, Bernardo M, Masana J, et al. Repetitive transcranial magnetic stimulation in the treatment of chronic negative schizophrenia: a pilot study. *J Neurol Neurosurg PSYCHIATRY*. 1999;67(1):129-130. doi:10.1136/jnnp.67.1.129

17. Cordes J, Falkai P, Guse B, et al. Repetitive transcranial magnetic stimulation for the treatment of negative symptoms in residual schizophrenia: rationale and design of a sham-controlled, randomized multicenter study. *Eur Arch PSYCHIATRY Clin Neurosci*. 2009;259:189-197. doi:10.1007/s00406-009-0060-y

18. Ćurčić-Blake B, Kos C, Aleman A. Causal connectivity from right DLPFC to IPL in schizophrenia patients: a pilot study. *Schizophr Heidelb*. 2022;8(1):16. doi:10.1038/s41537-022-00216-0

19. D’Alfonso A.A.L., Aleman A., Kessels R.P.C., et al. Transcranial magnetic stimulation of left auditory cortex in patients with Schizophrenia: Effects on hallucinations and neurocognition. *J Neuropsychiatry Clin Neurosci*. 2002;14(1):77-79. doi:10.1176/jnp.14.1.77

20. de Jesus D.R., Gil A., Barbosa L., et al. A pilot double-blind sham-controlled trial of repetitive transcranial magnetic stimulation for patients with refractory schizophrenia treated with clozapine. *Psychiatry Res*. 2011;188(2):203-207. doi:10.1016/j.psychres.2010.11.022

21. Demirtas-Tatlidede A., Freitas C., Cromer J.R., et al. Safety and proof of principle study of cerebellar vermal theta burst stimulation in refractory schizophrenia. *Schizophr Res*. 2010;124(1-3):91-100. doi:10.1016/j.schres.2010.08.015

22. Dlabac-de Lange J.J., Liemburg E.J., Bais L., Renken R.J., Knegtering H., Aleman A. Effect of rTMS on brain activation in schizophrenia with negative symptoms: A proof-of-principle study. *Schizophr Res*. 2015;168(1-2):475-482. doi:10.1016/j.schres.2015.06.018

23. Dlabac-de Lange J.J., Bais L., van Es F.D., et al. Efficacy of bilateral repetitive transcranial magnetic stimulation for negative symptoms of schizophrenia: results of a multicenter double-blind randomized controlled trial. *Psychol Med*. 2015;45(6):1263-1275. doi:10.1017/S0033291714002360

24. Dollfus S., Larmurier-Montagne A., Razafimandimby A., et al. Treatment of auditory hallucinations by combining high-frequency repetitive transcranial magnetic stimulation and functional magnetic resonance imaging. *Schizophr Res*. 2008;102(1-3):348-351. doi:10.1016/j.schres.2008.04.012

25. Dollfus S., Jaafari N., Guillin O., et al. High-Frequency Neuronavigated rTMS in Auditory Verbal Hallucinations: A Pilot Double-Blind Controlled Study in Patients With Schizophrenia. *Schizophr Bull*. 2018;44(3):505-514. doi:10.1093/schbul/sbx127

26. Eberle MC, Wildgruber D, Wasserka B, Fallgatter AJ, Plewnia C. Relief from chronic intractable auditory hallucinations after long-term bilateral theta burst stimulation. *Am J Psychiatry*. 2010;167(11):1410. doi:10.1176/appi.ajp.2010.10070988

27. Feinsod M., Kreinin B., Chistyakov A., Klein E. Preliminary evidence for a beneficial effect of low-frequency, repetitive transcranial magnetic stimulation in patients with major depression and schizophrenia. *Depress Anxiety*. 1998;7(2):65-68. doi:10.1002/%28SICI%291520-6394%281998%297:2%3C65::AID-DA2%3E3.0.CO;2-4

28. Fitzgerald P.B., Brown T.L., Marston N.A.U., et al. Reduced plastic brain responses in schizophrenia: A transcranial magnetic stimulation study. *Schizophr Res*. 2004;71(1):17-26. doi:10.1016/j.schres.2004.01.018

29. Fitzgerald P.B., Benitez J., Daskalakis J.Z., et al. A double-blind sham-controlled trial of repetitive transcranial magnetic stimulation in the treatment of refractory auditory hallucinations. *J Clin Psychopharmacol*. 2005;25(4):358-362. doi:10.1097/01.jcp.0000168487.22140.7f

30. Fitzgerald P.B., Herring S., Hoy K., et al. A study of the effectiveness of bilateral transcranial magnetic stimulation in the treatment of the negative symptoms of schizophrenia. *Brain Stimulat*. 2008;1(1):27-32. doi:10.1016/j.brs.2007.08.001

31. Francis M.M., Hummer T.A., Vohs J.L., et al. Cognitive effects of bilateral high frequency repetitive transcranial magnetic stimulation in early phase psychosis: a pilot study. *Brain Imaging Behav*. 2019;13(3):852-861. doi:10.1007/s11682-018-9902-4

32. Franck N., Poulet E., Terra J.-L., Dalery J., D’Amato T. Left temporoparietal transcranial magnetic stimulation in treatment-resistant schizophrenia with verbal hallucinations. *Psychiatry Res*. 2003;120(1):107-109. doi:10.1016/S0165-1781%2803%2900148-3

33. Gan H., Zhu J., Zhuo K., et al. High frequency repetitive transcranial magnetic stimulation of dorsomedial prefrontal cortex for negative symptoms in patients with schizophrenia: A double-blind, randomized controlled trial. *Psychiatry Res*. 2021;299((Gan, Zhuo, Xiang, Li, Zhu, Liu) First-episode Schizophrenia and Early Psychosis Program, Division of Psychotic Disorders, Shanghai Mental Health Center, Shanghai Jiao Tong University School of Medicine, Shanghai, China(Zhu, Tang, Qian, Wang) Department o):113876. doi:10.1016/j.psychres.2021.113876

34. Garg S., Goyal N., Tikka S.K., Sinha V.K. Exacerbation of auditory verbal hallucinations with adjunctive high-frequency cerebellar vermal repetitive transcranial magnetic stimulation in schizophrenia: A case report. *J ECT*. 2013;29(1):65-66. doi:10.1097/YCT.0b013e3182706aa1

35. Garg S., Sinha V.K., Tikka S.K., Mishra P., Goyal N. The efficacy of cerebellar vermal deep high frequency (theta range) repetitive transcranial magnetic stimulation (rTMS) in schizophrenia: A randomized rater blind-sham controlled study. *Psychiatry Res*. 2016;243((Garg, Mishra) Department of Psychiatry, Shri Guru Ram Rai Institute of Medical&Health Sciences, Dehradun, Uttarakhand, India(Sinha, Tikka, Goyal) KS Mani Center for Cognitive Neurosciences and Department of Psychiatry, Central Institute of Psychiatry,):413-420. doi:10.1016/j.psychres.2016.07.023

36. Geller V., Grisaru N., Abarbanel J.M., Lemberg T., Belmaker R.H. Slow magnetic stimulation of prefrontal cortex in depression and schizophrenia. *Prog Neuropsychopharmacol Biol Psychiatry*. 1997;21(1):105-110. doi:10.1016/S0278-5846%2896%2900161-3

37. Ghanbari Jolfaei A, Naji B, Nasr Esfehani M. Repetitive Transcranial Magnetic Stimulation in Resistant Visual Hallucinations in a Woman With Schizophrenia: A Case Report. *Iran J Psychiatry Behav Sci*. 2016;10(1):e3561. doi:10.17795/ijpbs-3561

38. Goyal N, Nizamie S, Desrkar P. Efficacy of adjuvant high frequency repetitive transcranial magnetic stimulation on negative and positive symptoms of schizophrenia: Preliminary results of a double-blind sham-controlled study. *J NEUROPSYCHIATRY Clin Neurosci*. 2007;19(4):464-467. doi:10.1176/appi.neuropsych.19.4.464

39. Goyal N., Vidya K.L., Sinha V.K. Priming rTMS for treatment resistant auditory hallucinations in schizophrenia. *J Neuropsychiatry Clin Neurosci*. 2015;27(2):e177-e178. doi:10.1176/appi.neuropsych.14080191

40. Gupta P, Sahu A, Prasad S, Sinha VK, Bakhla AK. Memory changes following adjuvant temporo-parietal repetitive transcranial magnetic stimulation in schizophrenia. *Indian J Psychiatry*. 2021;63(1):66-69. doi:10.4103/psychiatry.IndianJPsychiatry_532_20

41. Hajak G., Marienhagen J., Langguth B., Werner S., Binder H., Eichhammer P. High-frequency repetitive transcranial magnetic stimulation in schizophrenia: A combined treatment and neuroimaging study. *Psychol Med*. 2004;34(7):1157-1163. doi:10.1017/S0033291704002338

42. Hallmayer J. Repetitive transcranial magnetic stimulation over temporoparietal cortices in the treatment of refractory auditory hallucinations in patients with schizophrenia. *Curr Psychiatry Rep*. 2005;7(3):160-161. doi:10.1007/s11920-005-0048-x

43. Hoffman R.E., Boutros N.N., Berman R.M., et al. Transcranial magnetic stimulation of left temporoparietal cortex in three patients reporting hallucinated “voices.” *Biol Psychiatry*. 1999;46(1):130-132. doi:10.1016/S0006-3223%2898%2900358-8

44. Hoffman R, Boutros N, Hu S, Berman R, Krystal J, Charney D. Transcranial magnetic stimulation and auditory hallucinations in schizophrenia. *LANCET*. 2000;355(9209):1073-1075. doi:10.1016/S0140-6736(00)02043-2

45. Hoffman R.E., Hawkins K.A., Gueorguieva R., et al. Transcranial magnetic stimulation of left temporoparietal cortex and medication-resistant auditory hallucinations. *Arch Gen Psychiatry*. 2003;60(1):49-56. doi:10.1001/archpsyc.60.1.49

46. Hoffman R.E., Gueorguieva R., Hawkins K.A., et al. Temporoparietal transcranial magnetic stimulation for auditory hallucinations: Safety, efficacy and moderators in a fifty patient sample. *Biol Psychiatry*. 2005;58(2):97-104. doi:10.1016/j.biopsych.2005.03.041

47. Hoffman R.E., Wu K., Pittman B., et al. Transcranial magnetic stimulation of wernicke’s and right homologous sites to curtail voices: A randomized trial. *Biol Psychiatry*. 2013;73(10):1008-1014. doi:10.1016/j.biopsych.2013.01.016

48. Holi M.M., Eronen M., Toivonen K., Toivonen P., Marttunen M., Naukkarinen H. Left prefrontal repetitive transcranial magnetic stimulation in schizophrenia. *Schizophr Bull*. 2004;30(2):429-434. doi:10.1093/oxfordjournals.schbul.a007089

49. Horacek J., Brunovsky M., Novak T., et al. Effect of low-frequency rTMS on electromagnetic tomography (LORETA) and regional brain metabolism (PET) in schizophrenia patients with auditory hallucinations. *Neuropsychobiology*. 2007;55(3-4):132-142. doi:10.1159/000106055

50. Huang W, Shen F, Zhang J, Xing B. Effect of Repetitive Transcranial Magnetic Stimulation on Cigarette Smoking in Patients with Schizophrenia. *Shanghai Arch Psychiatry*. 2016;28(6):309-317. doi:10.11919/j.issn.1002-0829.216044

51. Jandl M., Bittner R., Sack A., et al. Changes in negative symptoms and EEG in schizophrenic patients after repetitive Transcranial Magnetic Stimulation (rTMS): An open-label pilot study. *J Neural Transm*. 2005;112(7):955-967. doi:10.1007/s00702-004-0229-5

52. Jandl M, Steyer J, Weber M, et al. Treating auditory hallucinations by transcranial magnetic stimulation: A randomized controlled cross-over trial. *NEUROPSYCHOBIOLOGY*. 2006;53(2):63-69. doi:10.1159/000091721

53. Jin Y., Potkin S.G., Kemp A.S., et al. Therapeutic effects of individualized alpha frequency transcranial magnetic stimulation (alphaTMS) on the negative symptoms of schizophrenia. *Schizophr Bull*. 2006;32(3):556-561. doi:10.1093/schbul/sbj020

54. Kar S.K., Pahuja E. Repetitive transcranial magnetic stimulation for persistent auditory hallucination: Initial worsening of hallucination may not be a predictor of poor outcome. *Postepy Psychiatr Neurol*. 2016;25(4):251-254. doi:10.1016/j.pin.2016.11.002

55. Kim EJ, Yeo S, Hwang I, et al. Bilateral Repetitive Transcranial Magnetic Stimulation for Auditory Hallucinations in Patients with Schizophrenia: A Randomized Controlled, Cross-over Study. *Clin Psychopharmacol Neurosci*. 2014;12(3):222-228. doi:10.9758/cpn.2014.12.3.222

56. Kindler J., Homan P., Jann K., et al. Reduced neuronal activity in language-related regions after transcranial magnetic stimulation therapy for auditory verbal hallucinations. *Biol Psychiatry*. 2013;73(6):518-524. doi:10.1016/j.biopsych.2012.06.019

57. Kindler J., Homan P., Flury R., Strik W., Dierks T., Hubl D. Theta burst transcranial magnetic stimulation for the treatment of auditory verbal hallucinations: Results of a randomized controlled study. *Psychiatry Res*. 2013;209(1):114-117. doi:10.1016/j.psychres.2013.03.029

58. Klein E., Kolsky Y., Puyerovsky M., Koren D., Chistyakov A., Feinsod M. Right prefrontal slow repetitive transcranial magnetic stimulation in schizophrenia: A double-blind sham-controlled pilot study. *Biol Psychiatry*. 1999;46(10):1451-1454. doi:10.1016/S0006-3223%2899%2900182-1

59. Klirova M, Horacek J, Novak T, et al. Individualized rTMS neuronavigated according to regional brain metabolism ((18)FGD PET) has better treatment effects on auditory hallucinations than standard positioning of rTMS: a double-blind, sham-controlled study. *Eur Arch PSYCHIATRY Clin Neurosci*. 2013;263(6):475-484. doi:10.1007/s00406-012-0368-x

60. Koops S, van Dellen E, Schutte M, Nieuwdorp W, Neggers S, Sommer I. Theta Burst Transcranial Magnetic Stimulation for Auditory Verbal Hallucinations: Negative Findings From a Double-Blind-Randomized Trial. *Schizophr Bull*. 2016;42(1):250-257. doi:10.1093/schbul/sbv100

61. Kozak K, Sharif-Razi M, Morozova M, et al. Effects of short-term, high-frequency repetitive transcranial magnetic stimulation to bilateral dorsolateral prefrontal cortex on smoking behavior and cognition in patients with schizophrenia and non-psychiatric controls. *Schizophr Res*. 2018;197:441-443. doi:10.1016/j.schres.2018.02.015

62. Kumar N, Vishnubhatla S, Wadhawan AN, Minhas S, Gupta P. A randomized, double blind, sham-controlled trial of repetitive transcranial magnetic stimulation (rTMS) in the treatment of negative symptoms in schizophrenia. *Brain Stimulat*. 2020;13(3):840-849. doi:10.1016/j.brs.2020.02.016

63. Lee S.-H., Kim W., Chung Y.-C., et al. A double blind study showing that two weeks of daily repetitive TMS over the left or right temporoparietal cortex reduces symptoms in patients with schizophrenia who are having treatment-refractory auditory hallucinations. *Neurosci Lett*. 2005;376(3):177-181. doi:10.1016/j.neulet.2004.11.048

64. Levkovitz Y, Rabany L, Harel EV, Zangen A. Deep transcranial magnetic stimulation add-on for treatment of negative symptoms and cognitive deficits of schizophrenia: a feasibility study. *Int J Neuropsychopharmacol*. 2011;14(7):991-996. doi:10.1017/S1461145711000642

65. Li X., Yuan X., Kang Y., et al. A synergistic effect between family intervention and rTMS improves cognitive and negative symptoms in schizophrenia: A randomized controlled trial. *J Psychiatr Res*. 2020;126((Li, Yuan, Pang, Zhu, Song) The First Affiliated Hospital/Zhengzhou University, Zhengzhou, China(Li, Yuan, Pang, Zhu, Song) Biological Psychiatry International Joint Laboratory of Henan, Zhengzhou University, Zhengzhou, China(Li, Yuan, Pang, Zhu, Song) He):81-91. doi:10.1016/j.jpsychires.2020.04.009

66. Linsambarth S., Jeria A., Avirame K., Todder D., Riquelme R., Stehberg J. Deep Transcranial Magnetic Stimulation for the Treatment of Negative Symptoms in Schizophrenia: Beyond an Antidepressant Effect. *J ECT*. 2019;35(4):e46-e54. doi:10.1097/YCT.0000000000000592

67. Loo C.K., Sainsbury K., Mitchell P., Hadzi-Pavlovic D., Sachdev P.S. A sham-controlled trial of left and right temporal rTMS for the treatment of auditory hallucinations. *Psychol Med*. 2010;40(4):541-546. doi:10.1017/S0033291709990900

68. McIntosh A.M., Semple D., Tasker K., et al. Transcranial magnetic stimulation for auditory hallucinations in schizophrenia. *Psychiatry Res*. 2004;127(1-2):9-17. doi:10.1016/j.psychres.2004.03.005

69. Mendes-Filho V.A., de Jesus D.R., Belmonte-de-Abreu P., Cachoeira C.T., Rodrigues Lobato M.I. Effects of repetitive transcranial magnetic stimulation over supplementary motor area in patients with schizophrenia with obsessive-compulsive-symptoms: A pilot study. *Psychiatry Res*. 2016;242((Mendes-Filho, Belmonte-de-Abreu, Cachoeira, Rodrigues Lobato) Hospital de Clinicas de Porto Alegre (HCPA), Universidade Federal do Rio Grande do Sul, Porto Alegre (UFRGS), Rio Grande do Sul, Brazil(de Jesus) Centre for Addiction and Mental Health, Univer):34-38. doi:10.1016/j.psychres.2016.05.031

70. Mittrach M., Thunker J., Winterer G., et al. The tolerability of rTMS treatment in schizophrenia with respect to cognitive function. *Pharmacopsychiatry*. 2010;43(3):110-117. doi:10.1055/s-0029-1242824

71. Moeller SJ, Gil R, Weinstein JJ, et al. Deep rTMS of the insula and prefrontal cortex in smokers with schizophrenia: Proof-of-concept study. *Schizophr Heidelb*. 2022;8(1):6. doi:10.1038/s41537-022-00224-0

72. Mogg A., Purvis R., Eranti S., et al. Repetitive transcranial magnetic stimulation for negative symptoms of schizophrenia: A randomized controlled pilot study. *Schizophr Res*. 2007;93(1-3):221-228. doi:10.1016/j.schres.2007.03.016

73. Montagne-Larmurier A., Etard O., Razafimandimby A., Morello R., Dollfus S. Two-day treatment of auditory hallucinations by high frequency rTMS guided by cerebral imaging: A 6 month follow-up pilot study. *Schizophr Res*. 2009;113(1):77-83. doi:10.1016/j.schres.2009.05.006

74. Novak T., Horacek J., Mohr P., et al. The double-blind sham-controlled study of high-frequency rTMS (20Hz) for negative symptoms in schizophrenia: Negative results. *Neuroendocrinol Lett*. 2006;27(1-2):209-213.

75. Oh S.-Y., Kim Y.-K. Adjunctive treatment of bimodal repetitive transcranial magnetic stimulation (rTMS) in pharmacologically non-responsive patients with schizophrenia: A preliminary study. *Prog Neuropsychopharmacol Biol Psychiatry*. 2011;35(8):1938-1943. doi:10.1016/j.pnpbp.2011.07.015

76. Oxley T., Fitzgerald P.B., Brown T.L., De Castella A., Jeff Daskalakis Z., Kulkarni J. Repetitive transcranial magnetic stimulation reveals abnormal plastic response to premotor cortex stimulation in schizophrenia. *Biol Psychiatry*. 2004;56(9):628-633. doi:10.1016/j.biopsych.2004.08.023

77. Paillere-Martinot M.-L., Galinowski A., Plaze M., et al. Active and placebo transcranial magnetic stimulation effects on external and internal auditory hallucinations of schizophrenia. *Acta Psychiatr Scand*. 2017;135(3):228-238. doi:10.1111/acps.12680

78. Plewnia C., Zwissler B., Wasserka B., Fallgatter A.J., Klingberg S. Treatment of auditory hallucinations with bilateral theta burst stimulation: A randomized controlled pilot trial. *Brain Stimulat*. 2014;7(2):340-341. doi:10.1016/j.brs.2014.01.001

79. Poulet E., Brunelin J., Bediou B., et al. Slow transcranial magnetic stimulation can rapidly reduce resistant auditory hallucinations in schizophrenia. *Biol Psychiatry*. 2005;57(2):188-191. doi:10.1016/j.biopsych.2004.10.007

80. Poulet E., Brunelin J., Ben Makhlouf W., D’Amato T., Saoud M. A case report of cTBS for the treatment of auditory hallucinations in a patient with schizophrenia. *Brain Stimulat*. 2009;2(2):118-119. doi:10.1016/j.brs.2008.09.008

81. Prikryl R., Kasparek T., Skotakova S., Ustohal L., Kucerova H., Ceskova E. Treatment of negative symptoms of schizophrenia using repetitive transcranial magnetic stimulation in a double-blind, randomized controlled study. *Schizophr Res*. 2007;95(1-3):151-157. doi:10.1016/j.schres.2007.06.019

82. Prikryl R., Ustohal L., Prikrylova-Kucerova H., Cermakova I., Ceskova E. Effects of sequential frontotemporal repetitive transcranial magnetic stimulation (rTMS) on schizophrenia. *Act Nerv Super Rediviva*. 2010;52(1):37-41.

83. Prikryl R, Ustohal L, Prikrylova-Kucerova H, Ceskova E. Occurrence of robust psychotic symptoms after initial rTMS treatment session. *J ECT*. 2011;27(3):265-266. doi:10.1097/YCT.0b013e3181f665bc

84. Prikryl R., Ustohal L., Prikrylova Kucerova H., et al. A detailed analysis of the effect of repetitive transcranial magnetic stimulation on negative symptoms of schizophrenia: A double-blind trial. *Schizophr Res*. 2013;149(1-3):167-173. doi:10.1016/j.schres.2013.06.015

85. Prikryl R., Ustohal L., Kucerova H.P., et al. Repetitive transcranial magnetic stimulation reduces cigarette consumption in schizophrenia patients. *Prog Neuropsychopharmacol Biol Psychiatry*. 2014;49((Prikryl, Ustohal, Kucerova, Kasparek, Hublova, Vrzalova, Ceskova) CEITEC-Central European Institute of Technology, Masaryk University, Czechia(Prikryl, Ustohal, Kucerova, Kasparek, Vrzalova, Ceskova) Department of Psychiatry Faculty of Medicine, Masary):30-35. doi:10.1016/j.pnpbp.2013.10.019

86. Quan W.X., Zhu X.L., Qiao H., et al. The effects of high-frequency repetitive transcranial magnetic stimulation (rTMS) on negative symptoms of schizophrenia and the follow-up study. *Neurosci Lett*. 2015;584((Quan, Qiao, Zhang, Zhou, Wang) Institute of Mental Health, Peking University, 51 Hua Yuan Bei Road, Beijing 100191, China(Quan, Qiao, Zhang, Zhou, Wang) Key Laboratory of Mental Health, Ministry of Health, Institute of Mental Health, The Sixth Hospital,):197-201. doi:10.1016/j.neulet.2014.10.029

87. Rachid F., Vianin P., Besse C. Safety and efficacy of continuous theta burst stimulation at 30 Hz in a patient with chronic auditory hallucinations: A case study in a private practice setting. *Brain Stimulat*. 2013;6(4):707-708. doi:10.1016/j.brs.2013.01.006

88. Rollnik J.D., Huber T.J., Mogk H., et al. High frequency repetitive transcranial magnetic stimulation (rTMS) of the dorsolateral prefrontal cortex in schizophrenic patients. *NeuroReport*. 2000;11(18):4013-4015. doi:10.1097/00001756-200012180-00022

89. Rollnik J, Seifert J, Huber T, et al. Repetitive transcranial magnetic stimulation and electroconvulsive therapy in a patient with treatment-resistant schizoaffective disorder. *Depress ANXIETY*. 2001;13(2):103-104. doi:10.1002/da.1024

90. Rosa M.O., Gattaz W.F., Rosa M.A., et al. Effects of repetitive transcranial magnetic stimulation on auditory hallucinations refractory to clozapine. *J Clin Psychiatry*. 2007;68(10):1528-1532. doi:10.4088/JCP.v68n1009

91. Rosenberg O, Roth Y, Kotler M, Zangen A, Dannon P. Deep transcranial magnetic stimulation for the treatment of auditory hallucinations: a preliminary open-label study. *Ann Gen Psychiatry*. 2011;10(1):3. doi:10.1186/1744-859X-10-3

92. Saba G., Verdon C.M., Kalalou K., et al. Transcranial magnetic stimulation in the treatment of schizophrenic symptoms: A double blind sham controlled study. *J Psychiatr Res*. 2006;40(2):147-152. doi:10.1016/j.jpsychires.2005.02.008

93. Sachdev P., Loo C., Mitchell P., Malhi G. Transcranial magnetic stimulation for the deficit syndrome of schizophrenia: A pilot investigation. *Psychiatry Clin Neurosci*. 2005;59(3):354-357. doi:10.1111/j.1440-1819.2005.01382.x

94. Schonfeldt-Lecuona C., Gron G., Walter H., et al. Stereotaxic rTMS for the treatment of auditory hallucinations in schizophrenia. *NeuroReport*. 2004;15(10):1669-1673. doi:10.1097/01.wnr.0000126504.89983.ec

95. Sidhoumi D., Braha S., Bouaziz N., Brunelin J., Benadhira R., Januel D. Evaluation of the therapeutic effect of theta burst stimulation on drug-resistant auditory hallucinations in a schizophrenic patient and its impact on cognitive function and neuronal excitability: A case study. *Clin Neurophysiol*. 2010;121(5):802. doi:10.1016/j.clinph.2009.12.033

96. Singh S, Kumar N, Verma R, Nehra A. The safety and efficacy of adjunctive 20-Hz repetitive transcranial magnetic stimulation for treatment of negative symptoms in patients with schizophrenia: A double-blinded, randomized, sham-controlled study. *Indian J Psychiatry*. 2020;62(1):21-29. doi:10.4103/psychiatry.IndianJPsychiatry_361_19

97. Slotema C.W., Blom J.D., De Weijer A.D., et al. Can low-frequency repetitive transcranial magnetic stimulation really relieve medication-resistant auditory verbal hallucinations? Negative results from a large randomized controlled trial. *Biol Psychiatry*. 2011;69(5):450-456. doi:10.1016/j.biopsych.2010.09.051

98. Slotema C, Blom J, de Weijer A, Hoek H, Sommer I. Priming does not enhance the efficacy of 1 Hertz repetitive transcranial magnetic stimulation for the treatment of auditory verbal hallucinations: Results of a randomized controlled study. *Brain Stimulat*. 2012;5(4):554-559. doi:10.1016/j.brs.2011.10.005

99. Sommer I, de Weijer A, Daalman K, et al. Can fMRI-guidance improve the efficacy of rTMS treatment for auditory verbal hallucinations? *Schizophr Res*. 2007;93(1-3):406-408. doi:10.1016/j.schres.2007.03.020

100. Stanford A.D., Corcoran C., Bulow P., Bellovin-Weiss S., Malaspina D., Lisanby S.H. High-frequency prefrontal repetitive transcranial magnetic stimulation for the negative symptoms of schizophrenia: A case series. *J ECT*. 2011;27(1):11-17. doi:10.1097/YCT.0b013e3181f41ea3

101. Su X, Liu H, Wang X, et al. Neuronavigated Repetitive Transcranial Stimulation Improves Neurocognitive Functioning in Veterans with Schizophrenia: A Possible Role of BDNF Polymorphism. *Curr Neuropharmacol*. Published online 2022. doi:10.2174/1570159X20666220803154820

102. Su X, Zhao L, Shang Y, et al. Repetitive transcranial magnetic stimulation for psychiatric symptoms in long-term hospitalized veterans with schizophrenia: A randomized double-blind controlled trial. *Front Psychiatry*. 2022;13:873057. doi:10.3389/fpsyt.2022.873057

103. Su X, Wang X, Pan X, et al. Effect of repetitive transcranial magnetic stimulation in inducing weight loss in patients with chronic schizophrenia: a randomized, double-blind controlled 4-week study. *Curr Neuropharmacol*. Published online 2022. doi:10.2174/1570159X20666220524123315

104. Subramanian P., Burhan A. Worsening of “passivity” symptoms with low-frequency bilateral temporo-parietal repetitive transcranial magnetic stimulation used to treat refractory auditory hallucinations: A case report. *Schizophr Res*. 2010;116(2-3):291-292. doi:10.1016/j.schres.2009.10.027

105. Subramanian P, Burhan A, Pallaveshi L, Rudnick A. The experience of patients with schizophrenia treated with repetitive transcranial magnetic stimulation for auditory hallucinations. *Case Rep Psychiatry*. 2013;2013:183582. doi:10.1155/2013/183582

106. Sverak T., Prikryl R., Mayerova M., Anderkova L. Intensive high-frequency repetitive transcranial magnetic stimulation in schizophrenia a case study. *J ECT*. 2014;30(4):e51-e53. doi:10.1097/YCT.0000000000000178

107. Sverak T, Mayerova M, Obdrzalkova M, Ustohal L. Accelerated Repetitive Transcranial Magnetic Stimulation in the Treatment of Negative Symptoms of Schizophrenia An Open-Label Study. *J ECT*. 2022;38(2):E24-E25.

108. Thirthalli J, Bharadwaj B, Kulkarni S, Gangadhar B, Kharawala S, Andrade C. Successful use of maintenance rTMS for 8 months in a patient with antipsychotic-refractory auditory hallucinations. *Schizophr Res*. 2008;100(1-3):351-352. doi:10.1016/j.schres.2008.01.003

109. Tikka S.K., Haque Nizamie S., Venkatesh Babu G.M., Aggarwal N., Das A.K., Goyal N. Safety and efficacy of adjunctive T burst repetitive transcranial magnetic stimulation to right inferior parietal lobule in schizophrenia patients with first-rank symptoms a pilot, exploratory study. *J ECT*. 2017;33(1):43-51. doi:10.1097/YCT.0000000000000343

110. Tyagi P., Dhyani M., Khattri S., Tejan V., Tikka S.K., Garg S. Efficacy of intensive bilateral Temporo-Parietal Continuous theta-burst Stimulation for Auditory VErbal hallucinations (TPC-SAVE) in schizophrenia: A randomized sham-controlled trial. *Asian J Psychiatry*. 2022;74((Tyagi, Dhyani, Khattri, Tejan, Garg) Department of Psychiatry, Shri Guru Ram Rai Institute of Medical and Health Sciences, Uttarakhand, Dehradun 248001, India(Tikka) Department of Psychiatry, All India Institute of Medical Sciences (AIIMS), Telangana, Bi):103176. doi:10.1016/j.ajp.2022.103176

111. Van Lutterveld R., Koops S., Schutter D.J.L.G., et al. The effect of rTMS on auditory hallucinations: Clues from an EEG-rTMS study. *Schizophr Res*. 2012;137(1-3):174-179. doi:10.1016/j.schres.2012.01.010

112. Vercammen A., Knegtering H., Bruggeman R., et al. Effects of bilateral repetitive transcranial magnetic stimulation on treatment resistant auditory-verbal hallucinations in schizophrenia: A randomized controlled trial. *Schizophr Res*. 2009;114(1-3):172-179. doi:10.1016/j.schres.2009.07.013

113. Voineskos A.N., Blumberger D.M., Schifani C., et al. Effects of Repetitive Transcranial Magnetic Stimulation on Working Memory Performance and Brain Structure in People With Schizophrenia Spectrum Disorders: A Double-Blind, Randomized, Sham-Controlled Trial. *Biol Psychiatry Cogn Neurosci Neuroimaging*. 2021;6(4):449-458. doi:10.1016/j.bpsc.2020.11.011

114. Wagner E., Wobrock T., Kunze B., et al. Efficacy of high-frequency repetitive transcranial magnetic stimulation in schizophrenia patients with treatment-resistant negative symptoms treated with clozapine. *Schizophr Res*. 2019;208((Wagner, Strube, Schneider-Axmann, Falkai, Hasan) Department of Psychiatry and Psychotherapy, Klinikum der Universitat Munchen, University Hospital, LMU Munich, Germany(Wobrock, Kunze) Department of Psychiatry and Psychotherapy, Georg-August-University Go):370-376. doi:10.1016/j.schres.2019.01.021

115. Walther S., Kunz M., Muller M., et al. Single Session Transcranial Magnetic Stimulation Ameliorates Hand Gesture Deficits in Schizophrenia. *Schizophr Bull*. 2020;46(2):286-293. doi:10.1093/schbul/sbz078

116. Wang L., Li Q., Wu Y., et al. Intermittent theta burst stimulation improved visual-spatial working memory in treatment-resistant schizophrenia: A pilot study. *J Psychiatr Res*. 2022;149((Wang, Ji, Wu, Xiao, Hu, Chen, Wang) Department of Neurology, The First Affiliated Hospital of Anhui Medical University, Hefei, China(Wu, Ji, Wang) School of Mental Health and Psychological Sciences, Anhui Medical University, Hefei, China(Wang) Institute):44-53. doi:10.1016/j.jpsychires.2022.02.019

117. Wen N, Chen L, Miao X, et al. Effects of High-Frequency rTMS on Negative Symptoms and Cognitive Function in Hospitalized Patients With Chronic Schizophrenia: A Double-Blind, Sham-Controlled Pilot Trial. *Front Psychiatry*. 2021;12:736094. doi:10.3389/fpsyt.2021.736094

118. Wing V.C., Bacher I., Wu B.S., Daskalakis Z.J., George T.P. High frequency repetitive transcranial magnetic stimulation reduces tobacco craving in schizophrenia. *Schizophr Res*. 2012;139(1-3):264-266. doi:10.1016/j.schres.2012.03.006

119. Wobrock T, Guse B, Cordes J, et al. Left Prefrontal High-Frequency Repetitive Transcranial Magnetic Stimulation for the Treatment of Schizophrenia with Predominant Negative Symptoms: A Sham-Controlled, Randomized Multicenter Trial. *Biol PSYCHIATRY*. 2015;77(11):979-988. doi:10.1016/j.biopsych.2014.10.009

120. Xiu MH, Guan HY, Zhao JM, et al. Cognitive Enhancing Effect of High-Frequency Neuronavigated rTMS in Chronic Schizophrenia Patients With Predominant Negative Symptoms: A Double-Blind Controlled 32-Week Follow-up Study. *Schizophr Bull*. 2020;46(5):1219-1230. doi:10.1093/schbul/sbaa035

121. Yu H.-C., Liao K.-K., Chang T.-J., Tsai S.-J. Transcranial magnetic stimulation in schizophrenia [6]. *Am J Psychiatry*. 2002;159(3):494-495. doi:10.1176/appi.ajp.159.3.494

122. Zeeuws D, Santermans L, Baeken C, Vanderbruggen N. INTENSIVE rTMS APPLICATIONS IN DIFFICULT TO TREAT PSYCHIATRIC PATIENTS: SOME CASES. *Psychiatr Danub*. 2010;22:S135-S136.

123. Zhao S, Kong J, Li S, Tong Z, Yang C, Zhong H. Randomized controlled trial of four protocols of repetitive transcranial magnetic stimulation for treating the negative symptoms of schizophrenia. *Shanghai Arch Psychiatry*. 2014;26(1):15-21. doi:10.3969/j.issn.1002-0829.2014.01.003

124. Zhu L., Zhang W., Zhu Y., et al. Cerebellar theta burst stimulation for the treatment of negative symptoms of schizophrenia: A multicenter, double-blind, randomized controlled trial. *Psychiatry Res*. 2021;305((Zhu, Zhang, Zhu, Mu, Wang, Cai, Xie) Shanghai Mental Health Center, Shanghai Jiao Tong University School of Medicine, Shanghai, China(Zhu, Zhang, Zhu, Mu, Wang, Cai, Xie) Shanghai Center for Mental Disease Control and Prevention, Shanghai, China(Zhu, Zha):114204. doi:10.1016/j.psychres.2021.114204

125. Zhuo K, Tang Y, Song Z, et al. Repetitive transcranial magnetic stimulation as an adjunctive treatment for negative symptoms and cognitive impairment in patients with schizophrenia: a randomized, double-blind, sham-controlled trial. *Neuropsychiatr Treat*. 2019;15:1141-1150. doi:10.2147/NDT.S196086

126. Zhuo C, Tian H, Zhou C, et al. Transcranial direct current stimulation of the occipital lobes with adjunct lithium attenuates the progression of cognitive impairment in patients with first episode schizophrenia. *Front Psychiatry*. 2022;13:962918. doi:10.3389/fpsyt.2022.962918
